# Biochemical Characterization of a Non-G4-Type RNA Aptamer That Lights Up a GFP-like Fluorogenic Ligand

**DOI:** 10.3390/molecules30081777

**Published:** 2025-04-15

**Authors:** Shunsuke Abe, Shino Aburaya, Takaki Koyama, Takashi Usui, Junro Yoshino, Shigeyoshi Matsumura, Yoshiya Ikawa

**Affiliations:** 1 Graduate School of Science and Engineering, University of Toyama, Gofuku 3190, Toyama 930-8555, Japanyoshino@sci.u-toyama.ac.jp (J.Y.); smatsumu@sci.u-toyama.ac.jp (S.M.); 2 Graduate School of Pharma-Medical Sciences, University of Toyama, Sugitani 2630, Toyama 930-0152, Japan

**Keywords:** fluorescence, GFP mimic, non-G4 type, RNA structure, RNA aptamer

## Abstract

The 17-3 RNA aptamer recognizes DMHBI and induces its fluorescence. We showed that the 17-3 RNA aptamer predominantly induced emission of the phenolate form of DMHBI. We also demonstrated that the active structure of the minimal form of the 17-3 aptamer possessed three stem elements and two large loop elements, which we named Karashi and its sequence-optimized variant, Jigarashi, respectively. Chemical modification experiments suggested that the two loop regions formed tertiary interactions and/or non-Watson–Crick base pairs, and no remarkable structural alterations occurred upon DMHBI binding. AlphaFold3 also predicted a tertiary structure of the ligand-free form of Jigarashi RNA, which was consistent with the results of chemical modification experiments.

## 1. Introduction

A class of RNA aptamers targeting non- or weakly emissive dyes in their free states have attracted considerable attention because they often strongly induce emission of the target dyes [1,2,3,4,5]. Such aptamer RNA/dye ligand (serving as a fluorogen of the resulting complex) pairs are called fluorogenic RNA aptamers. Following several earlier studies to produce fluorogenic RNA aptamers [6,7], Jaffrey and coworkers generated Spinach RNA (a fluorogenic aptamer) recognizing DFHBI (3,5-difluoro-4-hydroxy-benzylidene imidazolinone), a derivative of the non-fluorescent molecule HBI (4-hydroxy-benzylidene imidazolinone) derived from the GFP chromophore [8,9]. The DFHBI/Spinach RNA complex demonstrated the potential utility of fluorogenic RNA aptamers for a wide range of bioscience and analytical science applications [1,2,3,4,5,9]. Based on the development of the DFHBI/Spinach pair, increasing numbers of fluorogenic RNA aptamers (non-emissive dye/aptamer RNA pairs) have been generated and their biological and analytical applications have expanded [1,2]. Among the fluorogenic RNA aptamers generated and characterized so far, the majority of them (Spinach, Broccoli, Chili, Corn, Beetroot, Mango I–IV, and Peach) form guanine quadruplex (G4) motifs at their fluorophore-binding sites [1,2,3,4,5]. These G4-type aptamers require K^+^ to induce fluorescence, as K^+^ plays a crucial role in the formation and stabilization of G4 structures. However, this K^+^ dependency may limit their biological applications under low-K^+^ conditions, such as in extracellular fluids. To overcome this potential limitation, non-G4-type aptamers such as DIR2s, Pepper, and Squash RNAs have also been developed and characterized [1,2,3,4,5]. Nevertheless, their repertoire remains limited, and expanding the pool of non-G4-type fluorogenic RNA aptamers is desirable.

DMHBI (3,5-dimethoxy-4-hydroxy-benzylidene imidazolinone, Figure S1A) belonging to the HBI family of GFP-like fluorogens has dimethoxy substituents in its phenol moiety in place of the difluoro substituents of DFHBI (Figure S1) [8]. In neutral buffer solution, DMHBI is present in equilibrium between the neutral phenol form and anionic phenolate form with a p*K*_a_ of 8.1 (Ref. [8], see also Figure S1A,B). The absorption spectra of the phenol and phenolate forms showed single broad peaks at 380 nm and 470 nm, respectively, and their pH-dependent equilibrium exhibited three isosbestic points at 260 nm, 284 nm, and 407 nm (Figure S1B). Jaffrey and coworkers isolated four distinct RNA sequences (2-4, 3-6, 13-2, and 17-3) as aptamers capable of inducing florescence of DMHBI [8]. One of these aptamers (13-2 RNA) was truncated, yielding 13-2min RNA (Figure S2A) [8]. Further characterization and optimization yielded an optimized 13-2min RNA, which was named Chili RNA (Figure S2A) [10,11,12].

**Figure 1:**
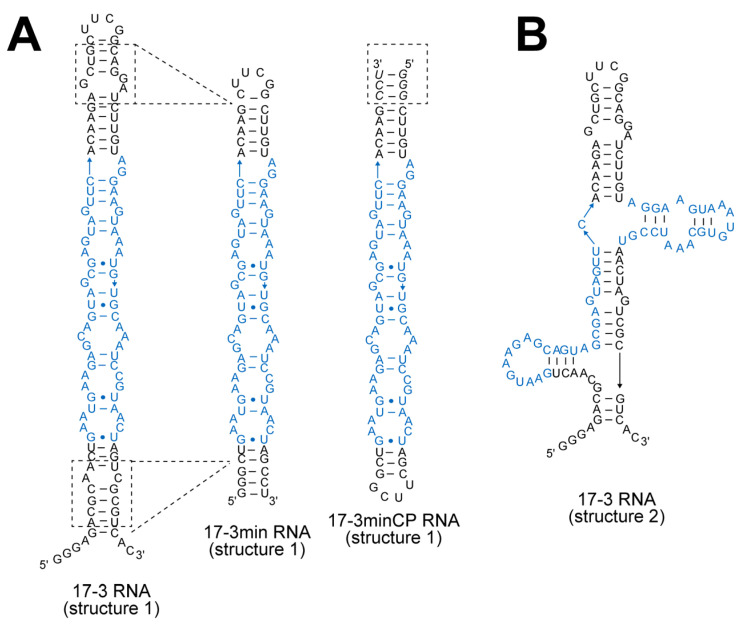
Sequences and predicted secondary structures of 17-3 RNA and its shortened variants. Mfold provided two predicted structures of 17-3 RNA (structure 1 and structure 2) with similar stability. Nucleotides shown in black are positions that were experimentally confirmed to form a stem or hairpin structure involved in structure 1 of 17-3 RNA. (**A**) Predicted structures of 17-3 RNA and its derivatives, all categorized under predicted structure 1. (**B**) Predicted structure 2 of 17-3 RNA.

Among the four fluorogenic aptamers for DMHBI (2-4, 3-6, 13-2, and 17-3), the excitation spectra indicated that emissions originated from excitation of the neutral phenol form of DMHBI in three complexes with 2-4 RNA, 3-6 RNA, and 13-2 RNA [8]. On the other hand, the excitation spectra of a complex of DMHBI with 17-3 RNA (Figure 1) showed emission with excitation at 485 nm [8], suggesting that 17-3 RNA can form a complex with the anionic phenolate form of DMHBI. Although the DMHBI/17-3 RNA complex has been applied as a signal output module in nucleic acid-based DNA sensing systems [13], its secondary and tertiary structures have not been investigated. Therefore, we performed biochemical characterization of 17-3 RNA. Through biochemical analysis of 17-3 RNA, we produced a truncated form (17-3min RNA), which enabled us to identify its active secondary structure. We named this new 17-3min RNA with the active secondary structure “Karashi RNA” after a hot Japanese mustard.

## 2. Results

### 2.1. Fluorescent Properties of the DMHBI/17-3 Complex

We first measured the absorption spectrum of DMHBI in the presence of 17-3 RNA. The DMHBI/17-3 complex showed two broad peaks at 414 nm and 477 nm (Figure S1C), which corresponded to the neutral and anionic forms of DMHBI, respectively. While the absorption maximum of the anionic form of DMHBI (477 nm) was similar to that of its free form (475 nm at pH 7.6) (Figure S1B), the absorption maximum of the neutral form (broad peak around 414 nm) was considerably red-shifted from that of the free ligand (380 nm) (Figure S1B,C). In pH 7.5 buffer, the emission spectrum of the DMHBI/17-3 complex excited at 400 nm was close to that reported previously [8] and showed a top peak at 547 nm (Figure S1D), which corresponded to absorption of the phenolate form of DMHBI. Excitation of the complex at 485 nm also induced emission with a broad peak at 533 nm (Figure S1E). This emission spectrum was similar but not identical to that of the complex excited at 400 nm (Figure S1D) because its emission peak was observed at 547 nm (Figure S1D). The fluorescence intensity (533 nm) with excitation at 485 nm was 2.5-fold higher than that (547 nm) with excitation at 400 nm (Figure S1D,E). The excitation spectra of the dye/RNA complex were measured at 550 nm (Figure S1F) and 530 nm (Figure S1G). A main peak at 482 nm was observed in both spectra but its intensity was much higher in the spectrum in which emission was detected at 530 nm. A small peak at 425 nm was observed only in the spectrum in which emission was detected at 550 nm (Figure S1G). Although further photophysical analysis is needed, similar but distinct spectra (peak top is 547 nm or 533 nm) of fluorescence excited at 400 nm or 485 nm may originate from a similar but distinct anionic form of a photoexcited DMHBI chromophore generated from the neutral ground-state form through the excited-state proton transfer (ESPT) process [12] or anionic ground-state form [8], respectively.

For comparison of emission induction of the neutral form of DMHBI, we confirmed fluorescence of the DMHBI/Chili complex in buffer containing KCl and Mg^2+^. With excitation at 485 nm, the DMHBI/Chili complex showed no fluorescence (Figure S2B). We then examined the emission of the DMHBI/Chili complex and DMHBI/17-3 complex at 535 nm with excitation at 400 nm and 360 nm. With excitation at 400 nm, both complexes showed enhanced emission at 535 nm (Figure S2C). The DMHBI/Chili complex showed a twofold higher emission than the DMHBI/17-3 complex did (Figure S2C). With excitation at 360 nm, 17-3 RNA hardly induced emission of DMHBI, whereas Chili RNA showed a modest (10-fold) enhancement in DMHBI fluorescence (Figure S2D). Based on these observations, we investigated the emission properties of the DMHBI/17-3 complex with excitation and emission wavelengths of 485 nm and 535 nm, respectively, because these wavelengths were characteristic of the DMHBI/17-3 RNA complex (Figure S2).

**Figure 2:**
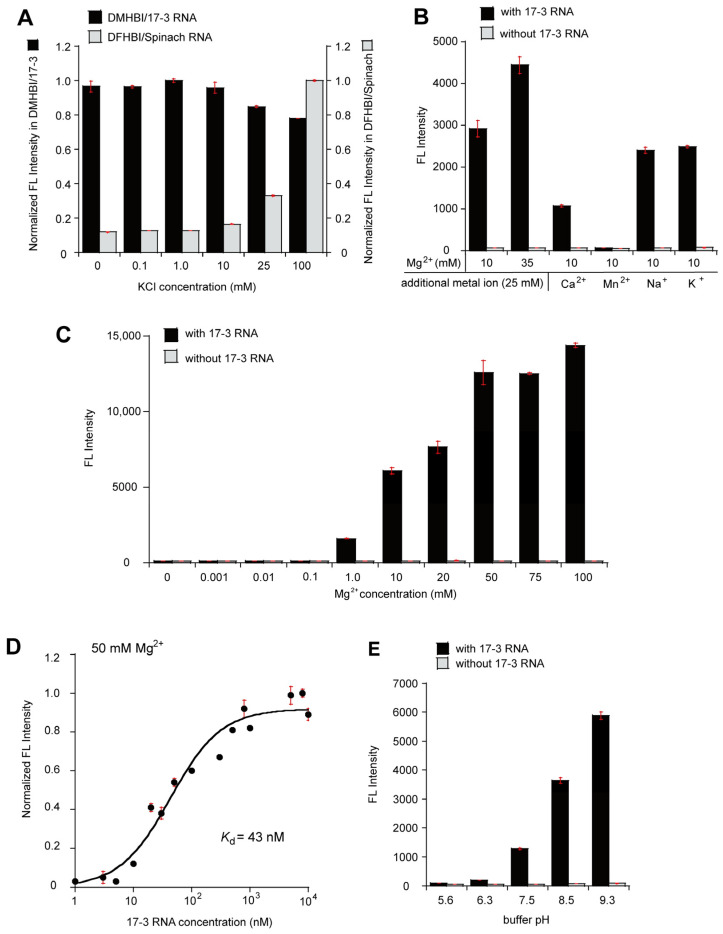
Fluorescent properties of the 17-3 RNA/DMHBI complex. (**A**) Effects of K^+^ on the fluorescence of the DMHBI/17-3 complex and DFHBI/Spinach complex. The concentrations of RNA, dye (HBI derivative), and Mg^2+^ are 0.1 μM, 0.1 μM, and 10 mM, respectively. (**B**) Effects of 25 mM divalent ions (Ca^2+^ or Mn^2+^) and 25 mM monovalent ions (Na^+^ or K^+^) on the DMHBI/17-3 complex supported with 10 mM Mg^2+^. The concentrations of RNA and DMHBI are both 0.1 μM. (**C**) Effects of Mg^2+^ concentration on the fluorescence of the DMHBI/17-3 complex. The concentrations of the 17-3 RNA and DMHBI are both 1.0 μM. (**D**) Estimation of the binding constant between 17-3 RNA and DMHBI in the presence of 50 mM Mg^2+^. The concentration of DMHBI is 5 nM. (**E**) Effects of buffer pH on the fluorescence of the DMHBI/17-3 complex. The concentrations of the 17-3 RNA, DMHBI, and Mg^2+^ are 5.0 μM, 1.0 μM, and 50 mM, respectively.

In the presence of 10 mM Mg^2+^, the emission of the DMHBI/17-3 complex was independent of KCl concentration (Figure 2A). This was in marked contrast to the emission of Spinach RNA (Figure 2A) and Chili RNA [10], in which G-quadruplex motifs were organized as binding sites for their cognate fluorophores. As the requirement for K^+^ is the most critical signature indicating that the function of a given aptamer RNA involves a G-quadruplex motif [14,15], this result strongly suggested that there is no G-quadruplex motif in the functional structure of 17-3 RNA. We next examined if a divalent ion (Ca^2+^ or Mn^2+^) other than Mg^2+^ can support the function of the DMHBI/17-3 complex. Neither Ca^2+^ nor Mn^2+^ supported efficient fluorescence of the complex (Figure S3). The function of Mg^2+^ (10 mM) in the formation of the emissive DMHBI/17-3 complex appeared to be inhibited by Mn^2+^ (25 mM) and Ca^2+^ (25 mM), while Na^+^ (25 mM) and K^+^ (25 mM) had no effect (Figure 2B).

The effects of Mg^2+^ concentration (0–100 mM) on the emission of the DMHBI/17-3 complex were investigated in the presence of equimolar amounts (1.0 μM each) of 17-3 RNA and DMHBI (Figure 2C). The emission of the DMHBI/17-3 complex increased with increasing Mg^2+^ concentration, showing 44-fold and 98-fold enhancements with 10 mM Mg^2+^ and 100 mM Mg^2+^, respectively (Figure 2C). A similar Mg^2+^-dependent enhancement in DMHBI fluorescence was observed in the presence of 1.0 μM RNA and an excess (10 μM) of DMHBI (Figure S3B). The critical dependence on Mg^2+^ for fluorescence induction by 17-3 RNA suggests that 17-3 RNA forms a tertiary structure and/or that Mg^2+^ directly mediates the interaction between DMHBI and the aptamer RNA.

We then evaluated the *K*_d_ between 17-3 RNA and DMHBI in the presence of 50 mM Mg^2+^ (Figure 2D). In the presence of 5 nM DMHBI, fluorescence-enhancement reached a maximum around 1.0 μM RNA and the *K*_d_ value was 43 nM (Figure 2D). The *K*_d_ values of the complex were 67 nM with 5 mM Mg^2+^ (Figure S3C) and 442 nM with 1 mM Mg^2+^ (Figure S3D). To further confirm whether 17-3 RNA recognized the phenolate form of DMHBI and induced emission, we altered the relative ratio of the neutral and phenolate forms in the solution by varying the solution pH (Figure 2E). The emission increased gradually with increasing pH (Figure 2E), suggesting that the emission originated from the phenolate form. *K*_d_ values between DMHBI and 17-3 RNA are relatively close between 5 mM Mg^2+^ (67 nM) and 50 mM Mg^2+^ (43 nM), while the fluorescence intensity of the complex increases in a Mg^2+^-dependent manner up to 100 mM (Figure 2C). This apparent discrepancy may suggest that, after saturation of the DMHBI/17-3 RNA complex formation, Mg^2+^ contributes to fine-tuning the structure of the complex. Since the fluorescence observed in this study mainly arises from the phenolate form of DMHBI, Mg^2+^ may interact with the phenolate moiety to enhance fluorescence from the dye/RNA complex.

### 2.2. Identification of the Essential Elements of 17-3 RNA to Generate Karashi RNA

To elucidate the essential elements and secondary structure of the 17-3 RNA responsible for the induction of DMHBI fluorescence, we predicted its secondary structure using mfold and MXfold2 [16,17,18]. Prediction with mfold afforded two structures; one was an extended structure (structure 1, Figure 1A Left) and the other had two sets of three-way junctions (structure 2, Figure 1B). Prediction with MXfold2 afforded a structure similar to structure 2 produced by mfold (Figure S4). We first examined the truncation of the 17-3 RNA based on structure 1 (Figure 1A) because the top and bottom stems of this structure seemed stable. The bottom element was truncated using the parent 17-3 RNA, while truncation of the top elements was performed using a circularly permuted form of the 17-3 RNA (Figure S5A). Truncation of the two elements provided shortened forms of the 17-3 RNA without disturbing its fluorescent properties (17-3min and 17-3minCP, Figure 1A and Figure S5C). One of the shortened variants (17-3min, Figure 1A) was mainly used for subsequent analysis. Based on the experimental confirmation that the 17-3min RNA retained the emission properties, we again predicted its secondary structure.

**Figure 3:**
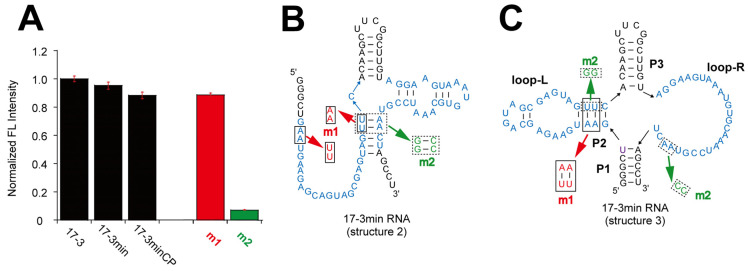
Truncation of the 17-3 RNA and evaluation of its functional secondary structure. (**A**) The fluorescence of the shortened forms (17-3min and 17-3minCP) and two mutants (m1 and m2) of 17-3 RNA. The concentrations of RNA, DMHBI, and Mg^2+^ are 0.1 μM, 0.1 μM, and 10 mM, respectively. (**B**) Predicted structure 2 of 17-3min RNA. (**C**) Predicted structure 3 of 17-3min RNA.

**Figure 4:**
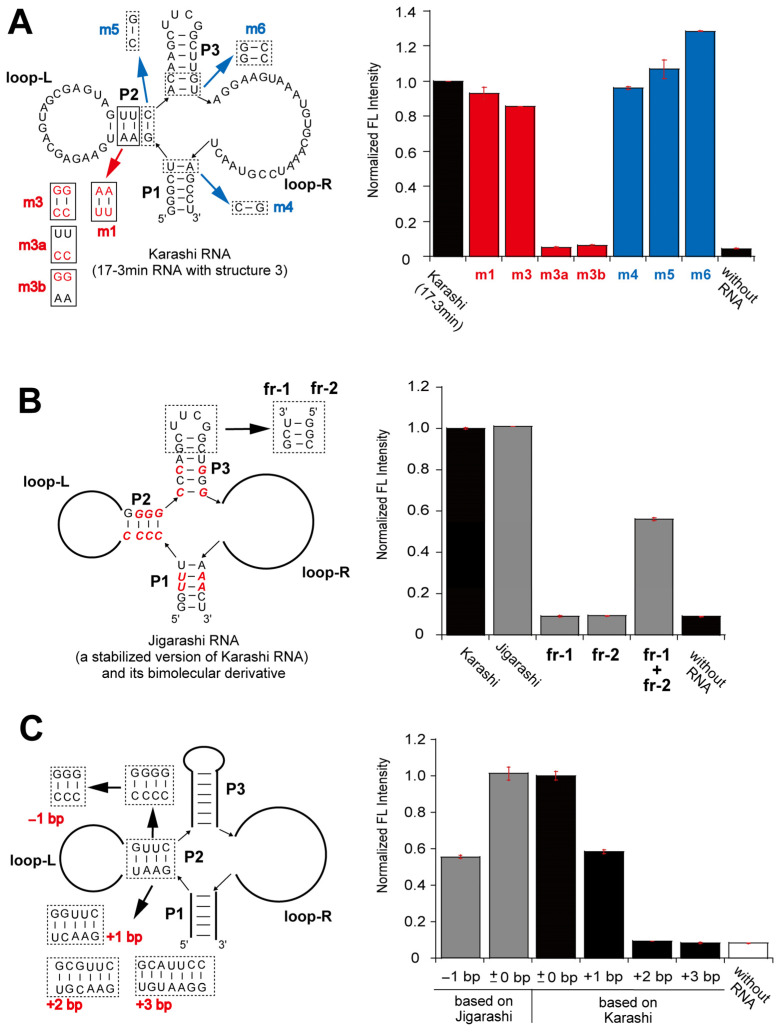
Evaluation of structure 3 of 17-3min RNA (Karashi RNA). (**A**) Evaluation of three stem elements in structure 3. The concentrations of RNA, DMHBI, and Mg^2+^ are 0.1 μM, 0.1 μM, and 10 mM, respectively. (**B**) The reconstitution of Jigarashi RNA (a stabilized version of Karashi RNA) from two RNA fragments containing loop-L and loop-R elements. The concentrations of each RNA, DMHBI, and Mg^2+^ are 0.1 μM, 0.1 μM, and 10 mM, respectively. (**C**) Effects of the length of P2 elements on the Karashi/DMHBI complex (or Jigarashi/DMHBI complex). The concentrations of RNA, DMHBI, and Mg^2+^ are 0.1 μM, 0.1 μM, and 10 mM, respectively.

The truncated form of structure 1 (Figure 1A right) was predicted by mfold as a single possible structure. We also manually adapted structure 2 of the parent 17-3 RNA to the 17-3min variant (Figure 3B). Prediction with MXfold2, however, provided a structure that was distinct from structures 1 and 2 (Figure 3C). This structure (structure 3) had a stem-loop and a large loop on its left side (loop-L) and right side (loop-R), respectively (Figure 3C). We validated structure 1 through base substitution(s) or a single base deletion, which were designed to preserve or stabilize the structure (Figure S6A). Most of these mutations, however, abolished the emission properties of the parent 17-3min RNA (Figure S6B). These results suggested that structure 1 did not represent the actual structure. We next validated structure 2 and structure 3 simultaneously by replacing base pairs seemingly critical to one of the two structures (Figure 3B,C)—mutations in the m1 mutant would disrupt structure 2 but maintain structure 3 (Figure 3B,C). Mutations in the m2 mutant would retain structure 2 but disrupt structure 3 (Figure 3B,C). In fluorescence measurement of the dye/RNA complexes, the m1 mutant supported emission comparable to 17-3min RNA, while the m2 mutant did not induce the fluorescence of DMHBI (Figure 3A). Based on the results of these mutational analyses, we provisionally concluded that structure 3 (Figure 3C and Figure 4A) represented the active structure of 17-3min RNA. Preliminary mutation analysis suggested that the two base pairs on the left side of the structure predicted by MXfold2 were unlikely to be formed in the functional 17-3min RNA (Figure S7A).

To further validate structure 3, we performed compensatory mutation analysis of the possible duplex element (P2) in its left side elements (Figure 4A). Two base pair substitutions (m1 and m3) preserved induction of DMHBI fluorescence, while the disruption of base pairs in the stem element (m3a and m3b) abolished the fluorescence of the complex (Figure 4A). In this stage, we named the 17-3min RNA-forming structure 3 “Karashi”. To evaluate the junction region assembling three stems and loop-R, we examined the importance of identity of base pairs at the edges of the three stems (Figure 4A). Three mutants (m4, m5, and m6) showed that the base pair at the edge of P1 and P3 elements could be altered (Figure 4A). We then confirmed the active structure of Karashi (17-3min) RNA by designing a reconstitution system through splitting structure 3 at the UUCG loop in the P3 element (Figure 4B). Preliminary dissection and reconstitution of the 17-3min RNA, however, were unsuccessful, presumably because one or both fragments folded into alternative secondary structures to prevent their assembly. Therefore, we optimized three stem elements to avoid the formation of alternative structures and yield a stabilized variant of Karashi RNA [19], which was named Jigarashi (a regional variation of Karashi mustard) (Figure 4B). The Jigarashi RNA induced the emission of DMHBI and its fragments (fr-1 RNA and fr-2 RNA) assembled together to induce the emission of DMHBI (Figure 4B). It should also be noted that, in the presence of each fragment RNA at 0.8 μM, the emission intensity of the fr-1 RNA + fr-2 RNA complex became comparable to the parent Jigarashi RNA (0.8 μM) (Figure S7B). These experiments supported the validity of structure 3. In addition to the base pair identity of the P2 element (Figure 4A,B), we investigated the number of base pairs in P2. We extended the P2 element of Karashi RNA by adding one to three base pairs and also shortened the P2 element of the Jigarashi RNA by removing one base pair (Figure 4C). Variant RNAs with short (−1 bp) and extended (+1 bp) P2 stems partially retained fluorescence induction activity, although the levels of emission of these complexes were approximately half of the parent RNAs (Figure 4C). Variant RNAs with six and seven base pair P2 stems showed no fluorescence induction (Figure 4C).

**Figure 5:**
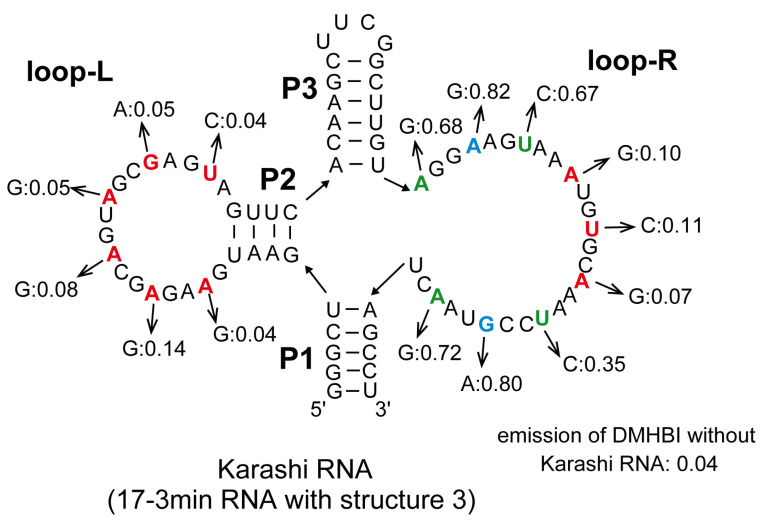
The effects of transition mutations in loop-L and loop-R regions of Karashi RNA. Nucleotides shown as red, green, or blue indicated that the relative fluorescent ability of each mutant possessing the transition mutation in the corresponding position was 0–0.2, 0.2–0.8, or 0.8–1.0 of that of the parent Karashi RNA, respectively. The concentrations of RNA, DMHBI, and Mg^2+^ are 0.1 μM, 0.1 μM, and 10 mM, respectively.

A series of mutational analyses strongly suggested that structure 3 reflects the secondary structure of Karashi RNA. To obtain information regarding the structure–function relationship of the loop elements on the left and right sides, we introduced a transition mutation (A > G, G > A, C > U, or U > C) to every two nucleotides in each region to prepare a total of 15 mutants (Figure 5). This analysis suggested that loop-L was less tolerant to transition mutations than loop-R (Figure 5). It should also be noted that no mutants improved emission induction and only two mutants (A51G and G69A) retained 80% of the emission induction activity of the parent 17-3 (Figure 5).

### 2.3. Chemical Probing of Karashi (17-3min) RNA and Its Circular Permutant

**Figure 6:**
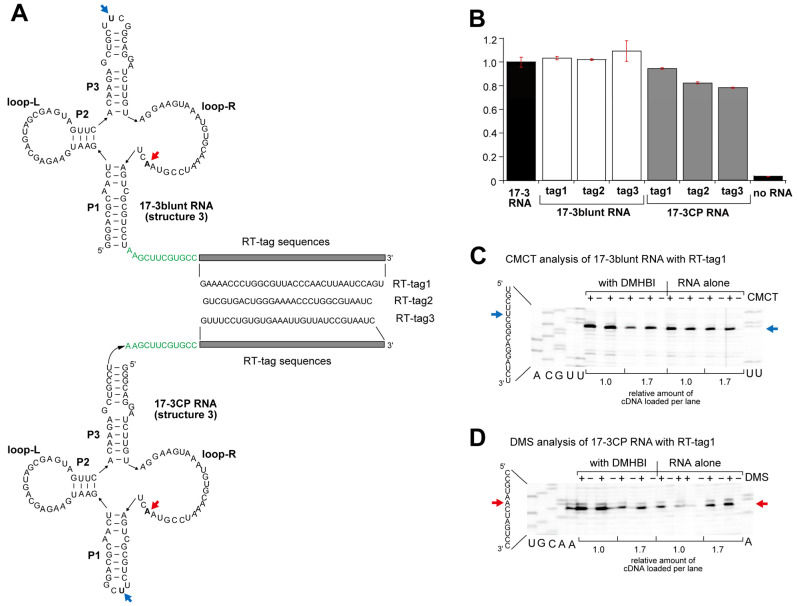
Derivatives of Karashi (17-3min) RNA designed for chemical modification experiments. (**A**) Three distinct RT-tag sequences were attached to the 3′ end of the 17-3blunt RNA and 17-3CP RNA. Red and blue arrows indicate the nucleotides that were modified most strongly with CMCT and DMS, respectively. Nucleotides shown in green indicate the linker region between the 17-3blunt or 17-3CP RNA and RT-tag. (**B**) Fluorescent properties of the 17-3blunt RNA and 17-3CP RNA were preserved in their derivatives possessing RT-tag sequences. (**C**) The blue arrow indicates the nucleotide that was modified most strongly with CMCT (top) in 17-3blunt RNA. (**D**) The red arrow indicates the nucleotide that was modified most strongly with DMS (bottom) in 17-3CP RNA.

To obtain structural information on Karashi (17-3min) RNA in DMHBI-free and DMHBI-bound forms, we performed chemical probing of the 17-3blunt RNA and its circularly permuted variant (17-3CP) (Figure 6A and Figure S5A) [20,21,22,23,24]. To detect chemically modified nucleotides through reverse transcription, we added primer-binding elements as tag sequences for reverse transcription (RT-tags) to the 17-3blunt RNA and 17-3CP RNA (Figure 6A). To avoid negative effects on the secondary structure and fluorescent properties of the parent RNAs, we prepared three different RT-tag sequences (Figure 6A). Fluorescent measurement indicated that three tag sequences did not disturb the fluorescent properties of the parent 17-3blunt RNA/DMHBI complex (Figure 6B). With DMHBI, the 17-3CP with appended RT-tags also showed comparable fluorescence to the parent 17-3CP RNA/DMHBI complex (Figure 6B).

**Figure 7:**
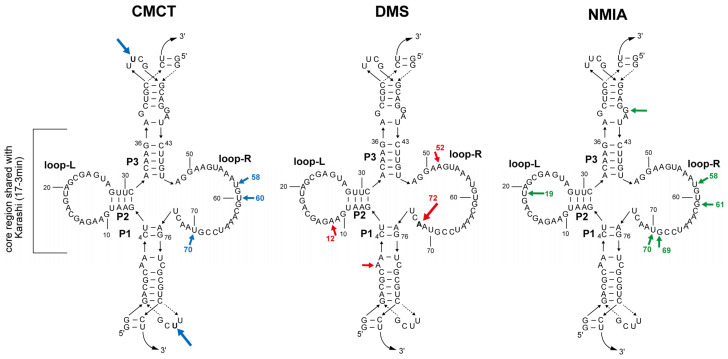
A summary of three chemical modification experiments for the core region of Karashi (17-3min). The modification with CMCT (**left**), DMS (**middle**), and NMIA (**right**). Arrows colored blue, red, or green indicate modifications with CMCT, DMS, or NMIA, respectively. The arrow size represents the degree of chemical modification. In the top and bottom regions of each secondary structure, solid and broken lines with small arrowheads indicate nucleotide sequences in the 17-3blunt RNA and 17-3CP RNA, respectively. Curved arrows indicate the 3′ ends of the 17-3blunt RNA and 17-3CP RNA, which are followed by RT-tag sequences.

We first performed chemical probing experiments using CMCT (*N*-cyclohexyl-*N*′-β-(4-methylmorpholinium) ethylcarbodiimide *p*-toluenesulfonate), which preferentially modifies the N3 position of U [25]. CMCT also modifies the N1 position of G less effectively than U [20,21,22]. Although no structural information of the 17-3 RNA including the base accessibility of CMCT was reported, the 17-3blunt RNA and 17-3CP RNA have a UUCG tetraloop that closes the top stem in the former and the bottom loop in the latter (Figure 6A and Figure S5A). These UUCG tetraloops can be used as positive controls to check the CMCT modification reaction (Figure 6C and Figure S8). With treatment of the RNA samples with CMCT, reverse transcription stopped at the second U in the UUCG loop (Figure 6C and Figure S8). This result was fully consistent with the results of previous studies in which the second U was strongly modified by CMCT [26] because this U was outside the tetraloop in the 3D structure [27]. In the sequence of the 17-3blunt RNA, no U or G residues were observed at which reverse transcription was stopped more strongly than the second U of the UUCG loop (Figure S8). Relatively strong stops occurred with three Us in the loop-R (corresponding to U58, U60, and U70 of the core region of the 17-3 RNA) (Figure 7 left and Figure S8). The modification pattern of the core region was highly similar between the parent 17-3 RNA and 17-3CP RNA (Figure S8).

We next performed chemical probing with DMS (dimethyl sulfate), which modifies the N1 position of A and the N1 position of C. In both the parent and CP forms, RNA samples treated with DMS yielded only three major stops, which corresponded to A12, A52, and A72 (Figure 7 middle and Figure S9). It should be noted that two sets of stretches of three As (55A-57A and 63A-65A) in the loop-Rs were hardly modified by DMS (Figure 7 middle and Figure S9). We then probed the 17-3blunt RNA and 17-3CP RNA with NMIA (*N*-methylisatoic anhydride), which modifies the 2′-OH group of the ribose moiety, and its extent of modification reflects the orientation and flexibility of 2′-OH groups in the given RNA structures [23,24]. In the modification patterns induced by NMINA, relatively strong modification sites were found at U19 in loop-L and four positions (U58, G61, G69, and U70) in loop-R (Figure 7 right and Figure S10). It should be noted that among the five positions modified, the extent of modification was strongest at U19 in loop-L. In the presence of 0.3 μM 17-3blunt RNA, we increased the amount of DMHBI from 0.3 μM (equimolar) to 0.9 M μM (threefold excess) but observed no significant changes in the modification patterns with CMCT, DMS, or NMIA (Figure S11).

### 2.4. Tertiary Structure Prediction of Karashi (17-3min) Aptamer by AlphaFold3

Although we performed biochemical characterization of Karashi (17-3min) and its parent 17-3 RNA, these experimental results were still insufficient in determining the tertiary structure. With advances in computer-assisted structural prediction of biomacromolecules, AlphaFold3 can be used to predict self-folding RNAs [28]. Therefore, we used AlphaFold3 to predict the tertiary structure of the Karashi RNA. We first predicted the structure of 17-3min RNA with the original P2 base pairs. The predicted structure, however, did not possess the P2 stem, and its 3D structure appeared based on a secondary structure resembling predicted structure 1 (Figure S12). Therefore, we altered the target RNA to Jigarashi RNA (Figure 4B) because the P2 stem of the variant is more thermodynamically stable than the parent Karashi RNA.

**Figure 8:**
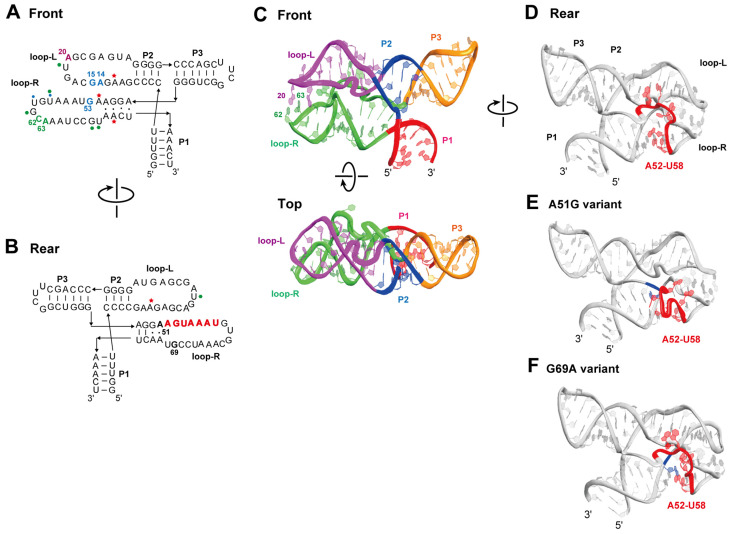
Three-dimensional structures of Jigarashi RNA and its variants predicted by AlphaFold3. (**A,B**) Secondary structures of Jigarashi RNA reflecting the front (**A**, **top**) and top (**A**, **bottom**) or rear (**B**) sides of its 3D structure predicted by AlphaFold3. Blue circles, red asterisks, and green hexagons indicate nucleotides modified by CMCT, DMS, and NMIA, respectively, in 17-3blunt RNA and 17-3CP RNA. The size of each symbol corresponds to the degree of modification. (**C**) The front of the 3D structure of Jigarashi RNA predicted by AlphaFold3. (**D**–**F**) The rear of AlphaFold3-predicted 3D structures of Jigarashi RNA (**C**), its A51G variant (**D**), and its G69A variant (**E**). Positions A52-U58 are shown in red. The A51G mutation (**D**) and G69A mutation (**E**) are shown in blue.

In the predicted structure of Jigarashi RNA, the P2 duplex was observed successfully and was stacked coaxially with the P3 duplex (Figure 8A). Therefore, we used this predicted structure to evaluate the 3D structure of the ligand-free state of the Karashi RNA in comparison with the biochemical results. The m3 mutant RNA also provided a tertiary structure highly similar to that of Jigarashi RNA. In addition to the coaxial stacking of P2 and P3 elements, an important feature of the predicted structure is the two loop regions (loop-L and loop-R), each of which seemed to form distorted helical structures containing multiple noncanonical base pairs. The two loops also appear to interact with each other through stacking interaction among A20 in loop-L and C62 and A63 in loop-R (Figure 8A). In addition to the three nucleotides (A20, C2, and A63), G53 in loop-R appears to form stacking interactions with A14 and G15 in loop-L (Figure 8B top, middle).

To evaluate the relationship between the predicted structure and functional activity, we also predicted two mutant aptamers (A51G and G69A), the fluorescent enhancement abilities of which were close to that of the parent Karashi RNA. Their predicted structures were compared with the parent Karashi RNA and also Jigarashi RNA. In the A51G mutant, the overall architecture was close to that of the parent aptamer. The coaxial stacking between P2 and P3 helices and the interloop interaction between C62, A63, and A20 were preserved. A significant structural difference was observed at the region of loop-R (A52-U58) where the interaction with loop-L was not observed in the A51G mutant without a significant reduction in fluorescent properties. The predicted structure of the G69A mutant was also close to that of the parent aptamer. While the conformation of the region of loop-R (A52-U58) was also similar to that of the parent aptamer, a possible stacking interaction among G53 in loop-R and A14 and G15 in loop-L seemed to not be formed, due to slight changes in the conformations of these regions (Figure S13). These structural differences among the parent RNA and its two single point mutants (A51G and G69A) suggested that positions G51-U58 form a single-stranded region with a relatively flexible confirmation. The biochemical results obtained in the present study were still insufficient in identifying the ligand-binding pocket, and the AlphaFold3-predicted structures were in the ligand-free state. The flexible structure of positions G51-U58 suggested that this region may be involved in recognition of the DMHBI fluorophore with an induced-fit mechanism.

## 3. Discussion

In this study, we characterized the 17-3 RNA aptamer to elucidate its secondary and tertiary structures through which it recognizes DMHBI dye as its target ligand and induces its emission. The fluorescence of DMHBI was induced by 17-3 RNA without K^+^, suggesting that the RNA recognizes DMHBI without the formation of a G-quadruplex motif. This is consistent with the sequence of the 17-3 RNA, which lacked consecutive G residues required for G-quadruplex motif formation. Truncation of the parent 17-3 RNA and mutation analysis revealed the functional secondary structure possessing two loop elements (loop-R and loop-L). This structure was predicted by neither mfold nor MXfold2 with the sequences of the parent 17-3 RNA, and the sequence of the shortened form (Karashi RNA) was needed to predict secondary structures close to the actual structure. These results indicated the importance of classical biochemical experiments in the study of RNA structure.

The predicted secondary structure was validated by chemical modification using three distinct reagents (CMCT, DMS, and NMIA). While the experimental results of chemical modification appeared consistent with the predicted secondary structure, the ligand-binding pocket could not be identified based on these data. Unexpectedly, loop-R and loop-L were resistant to modification, suggesting that these loops form non-Watson–Crick base pairs and/or tertiary interactions within each loop or between the two loops. This was supported by tertiary structure prediction by AlphaFold3 (Figure 8). Biochemical experiments indirectly played a key role in the accurate structural prediction by AlphaFold3. Without optimization of the primary sequence based on the biochemically defined secondary structure (Figure 4B), AlphaFold3 was unable to predict a tertiary structure that reflected the correct secondary structure (Figure S12). Among fluorogenic RNA aptamers for DMHBI, Chili RNA forms a typical G4 motif. In contrast, Karashi/Jigarashi RNAs are non-G4-type aptamers. A comparative study of Chili RNA and Karashi/Jigarashi RNAs would provide valuable insights into the design of fluorogenic RNA aptamers for DMHBI. A similar comparison between Spinach RNA and Squash RNA has already provided important insights into fluorogenic RNA aptamers for DFHBI [29].

The Karashi/Jigarashi RNAs characterized in this study contribute to expanding the repertoire of non-G4-type fluorogenic RNA aptamers that do not require K^+^ for their function. Although further characterization of its tertiary structure is needed, Karashi RNA may be a promising candidate as a fluorogenic aptamer for tracing extracellular RNAs (Ex RNAs), which are RNA molecules that can be isolated from extracellular fluids with low K^+^ concentration [30]. Non-G4-type RNA aptamers may also be promising for applications in eukaryotic cells, where RNA G-quadruplexes are reported to be globally unfolded [31]. The binding site for DMHBI in Karashi (17-3min) RNA also remains to be identified. Attempts to elucidate the structure by NMR and X-ray crystallography are now underway based on the results of biochemical analysis obtained in this study. For these structural studies, derivatives of 17-3 RNA generated in this study (Karashi RNA, Jigarashi RNA, and the bimolecular version) were employed. In parallel with the structural elucidation of Karashi RNA and its derivatives, including Jigarashi RNA, further optimization of Karashi RNA represents a promising approach to obtain variants with greater structural stability and functional efficiency. For these purposes, the droplet microfluidics technique provides a powerful platform for directed evolution and/or high-throughput screening of large pools of fluorescent aptamer variants based on their fluorescent enhancement capabilities [32,33]. We are now developing a droplet microfluidic-based screening system for improved variants of Karashi and Jigarashi aptamers. Since a water-in-oil droplet can be regarded as a quasi-cell-like compartment, improved variants of Krarashi/Jigarashi RNAs generated through in-droplet evolution may possess properties suitable for intracellular applications.

## 4. Materials and Methods

### 4.1. Chemicals

Unmodified DNA oligonucleotides were purchased from Eurofins DNA Synthesis (Tokyo, Japan). IRD700-labeled DNA oligonucleotides were purchased from LI-COR Biosciences (Lincoln, NE, USA). DMHBI was synthesized according to the procedure in the literature [8].

### 4.2. RNA Preparation

Double-stranded DNA templates for transcription of the 17-3 RNA and its derivatives, including Karashi (17-3min) RNA, were prepared by PCR with respective sets of oligonucleotides (a template and a pair of primers). The following are the sets of oligonucleotides we used for the 17-3 RNA and Karashi (17-3min) RNA. For the 17-3 RNA, we used 5′-GACGCAACTGAATGAAGAGCAGTAGCGAGTAGTTCACAAGAGCTGCTTCGGCAGGATCTTGTAGGAAGTAAATGTGCAAATCCGTAACTAGTCGCGTC-3′ (98 nucleotides) as the template and a pair of primers (5′-CTAATACGACTCACTATAGGGAGACGCAACTGAATGAAGAGCAGTAGC-3′ (48 nucleotides) as the sense primer where the T7 promoter sequence is underlined and 5′-GTGACGCGACTAGTTACGGATTTGCAC-3′ (27 nucleotides) as the antisense primer). For Karashi (17-3min) RNA, we used a set of oligonucleotides for Karashi (17-3min) RNA: 5′-GCTAGTTACGGATTTGCACATTTACTTCCTACAAGCCGAAGCTTGTGAACTACTCGCTACTGCTCTTCATTCAGC-3′ (75 nucleotides) as the template and a pair of primers (5′-CTAATACGACTCACTATAGGGCTGAATGAAGAGCAGTAGC-3′ (41 nucleotides) as the sense primer where the T7 promoter sequence is underlined and 5′-AGGCTAGTTACGGATTTGCAC-3′ (21 nucleotides) as the antisense primer). Transcription was performed with T7 RNA polymerase, and the desired RNA product was isolated by electrophoresis on 9% polyacrylamide gels (acrylamide/bisacrylamide 29:1) containing 8 M urea. The concentrations of the resulting RNA solutions were determined from the absorption at 260 nm (A_260_).

### 4.3. Standard Assay Conditions for the Fluorescence of DMHBI Complexed with the 17-3 RNA and Its Derivatives

An aqueous solution containing the 17-3 RNA or its derivative was heated to 80 °C for 2 min and cooled to 37 °C. To this solution were added 5× concentrated buffer and DMHBI solution. The resulting solution contained given concentrations of RNA, DMHBI, and MgCl_2_ in a 40 mM Tris-Cl (pH 7.5) buffer. The resulting solution was incubated for 5 min at 37 °C, and then fluorescence emission of the solution was measured using a plate reader Infinite F200-pro (Tecan, Männedorf, Switzerland) or BioTek Synergy H1(Agilent Technology, Santa Clare, CA, USA). The standard excitation and emission wavelengths we used in this study were 485 nm and 535 nm, respectively. All assays were repeated at least twice. The mean values are shown in the figures, in which error bars represent the range from the minimal to the maximal values. To determine the dissociation constant between 17-3 RNA and DMHBI, a titration experiment was performed according to a published method [34] with the following modification: a titration of 5 nM DMHBI was carried out with increasing concentrations of RNA, instead of 2 nM RNA with increasing concentrations of fluorophore as described in the original method [34]. The resulting data points were then fitted to a curve based on the Hill equation [34].

### 4.4. Chemical Modification of 17-3blunt RNA and 17-3CP RNA with CMCT

To 40 μL of a buffer solution (50 mM Tris-Cl pH 7.5 and 10 mM MgCl_2_) containing pre-folded 17-3blunt RNA (0.3 μM) or 17-3CP RNA (0.3 μM) with/without DMHBI (0.3 μM or 0.9 μM), 1.0 μL of 0.5 M CMCT in H_2_O was added, and the resulting mixture was incubated for 20 min at 37 °C. Modification reactions were stopped with 4.7 μL of 3 M NaOAc (pH 5.2). After treatment with phenol and diethyl ether, RNA was precipitated by adding 170 μL of EtOH. CMCT-treated RNA was dissolved in 10 μL of H_2_O.

### 4.5. Chemical Modification of 17-3blunt RNA and 17-3CP RNA with DMS

To 40 μL of a buffer solution (50 mM Tris-Cl pH 7.5 and 10 mM MgCl_2_) containing pre-folded 17-3blunt RNA (0.3 μM) or 17-3CP RNA (0.3 μM) with/without DMHBI (0.3 μM or 0.9 μM), 1.0 μL of 10% DMS in ethanol was added and the resulting mixture was incubated for 5 min at 37 °C. Modification reactions were stopped with 10 μL of 1 M aqueous solution of 2-mercaptoethanol and 5.7 μL of 3 M NaOAc (pH 7.0). After treatment with phenol and diethyl ether, RNA was precipitated by adding 170 μL of EtOH. DMS-treated RNA was dissolved in 10 μL of H_2_O.

### 4.6. Chemical Modification of 17-3blunt RNA and 17-3CP RNA with NMIA

To 40 μL of a buffer solution (50 mM Tris-Cl pH 7.5 and 10 mM MgCl_2_) containing pre-folded 17-3blunt RNA (0.3 μM) or 17-3CP RNA (0.3 μM) with/without DMHBI (0.3 μM or 0.9 μM), 1.0 μL of 34 mM NMIA in H_2_O was added and the resulting mixture was incubated for 45 min at 37 °C. Modification reactions were stopped with 4.9 μL of 3 M NaOAc (pH 7.0). After treatment with phenol and diethyl ether, RNA was precipitated by adding 170 μL of EtOH. NMIA-treated RNA was dissolved in 10 μL of H_2_O.

### 4.7. Reverse Transcription of Chemically Modified RNAs and Electrophoresis of the Resulting cDNAs

An aqueous solution containing RNA modified chemically with CMCT, DMS, or NMIA was subjected to reverse transcription with ReverTra Ace (Toyobo, Osaka, Japan) with the IRD700-labeled DNA primer (5′-GATTAAGTTGGGTAACGCCAGGGTTTTC-3′ as RT-tag1; 5′-GATTACGCCAGGGTTTTCCCAGTCACGAC-3′ as RT-tag2; or 5′-CGGATAACAATTTCACACAGGAAAC-3′ as RT-tag3). The resulting cDNAs were electrophoresed and analyzed using a DNA Analyzer Model 4300 (LI-COR Biosciences, Lincoln, NE, USA).

## 5. Conclusions

We characterized 17-3 RNA and identified the active form of its core elements, which was named Karashi RNA. The biochemical and structural properties of Karashi RNA were distinct and may be complementary to Chili RNA, although they both recognize DMHBI and induce its emission. Although the binding pocket for DMHBI has yet to be identified, our study will contribute to the further improvement of RNA aptamers that can light up DMHBI dye through rational and/or guided evolutionary approaches.

## Data Availability

Data are contained within the article and Supplementary Materials.

## Supplementary Materials

The following supporting information can be downloaded at https://www.mdpi.com/article/10.3390/molecules30081777/s1. Figure S1: Photophysical properties of DMHBI and DMHBI/17-3 complex; Figure S2: Comparison of fluorescent properties of DMHBI/17-3 RNA complex with DMHBI/Chili complex; Figure S3: Fluorescent properties of the DMHBI/17-3 complex; Figure S4: Secondary structure of 17-3 RNA predicted by MXFold2; Figure S5: Truncation of 17-3 RNA; Figure S6: Evaluation of predicted structure 1 through base substitution mutations; Figure S7: Evaluation of structure 3 of 17-3min RNA, named Karashi RNA; Figure S8: CMCT modification of 17-3blunt and 17-3CP; Figure S9: DMS modification of 17-3blunt and 17-3CP; Figure S10: NMIA modification of 17-3blunt and 17-3CP; Figure S11: The chemical modification of 17-3blunt RNA (0.3 μM) in the presence of an equimolar amount (0.3 μM) or a three-fold excess (0.9 μM) of DMHBI; Figure S12: A 3D structure of 17-3min RNA predicted by AlphaFold3; Figure S13: Three-dimensional structures of Jigarashi RNA and its variants predicted by AlphaFold3.

## Abbreviations

The following abbreviations are used in this manuscript:

DFHBI	3,5-difluoro-4-hydroxy-benzylidene imidazolinone
HBI	4-hydroxy-benzylidene imidazolinone
DMHBI	3,5-dimethoxy-4-hydroxy-benzylidene imidazolinone
CMCT	*N*-cyclohexyl-*N*′-β-(4-methylmorpholinium) ethylcarbodiimide *p*-toluenesulfonate
DMS	dimethyl sulfate
NMIA	*N*-methylisatoic anhydride
