# Supplementary material for: Biochemical Characterization of a Non-G4-Type RNA Aptamer That Lights Up a GFP-like Fluorogenic Ligand
Source: Molecules. 2025 Apr 15;30(8):1777. doi: 10.3390/molecules30081777 (PMC12029786; doi:10.3390/molecules30081777)
Supplement: Supplementary file 1 [file molecules-30-01777-s001.zip › molecules-3567969-supplementary.pdf]

Supporting Information for

Biochemical characterization of a non-G4 type RNA aptamer that lights up a GFP-like fluorogenic ligand

Shunsuke Abe, Shino Aburaya, Takaki Koyama, Takashi Usui, Junro Yoshino, Shigeyoshi Matsumura. and Yoshiya Ikawa

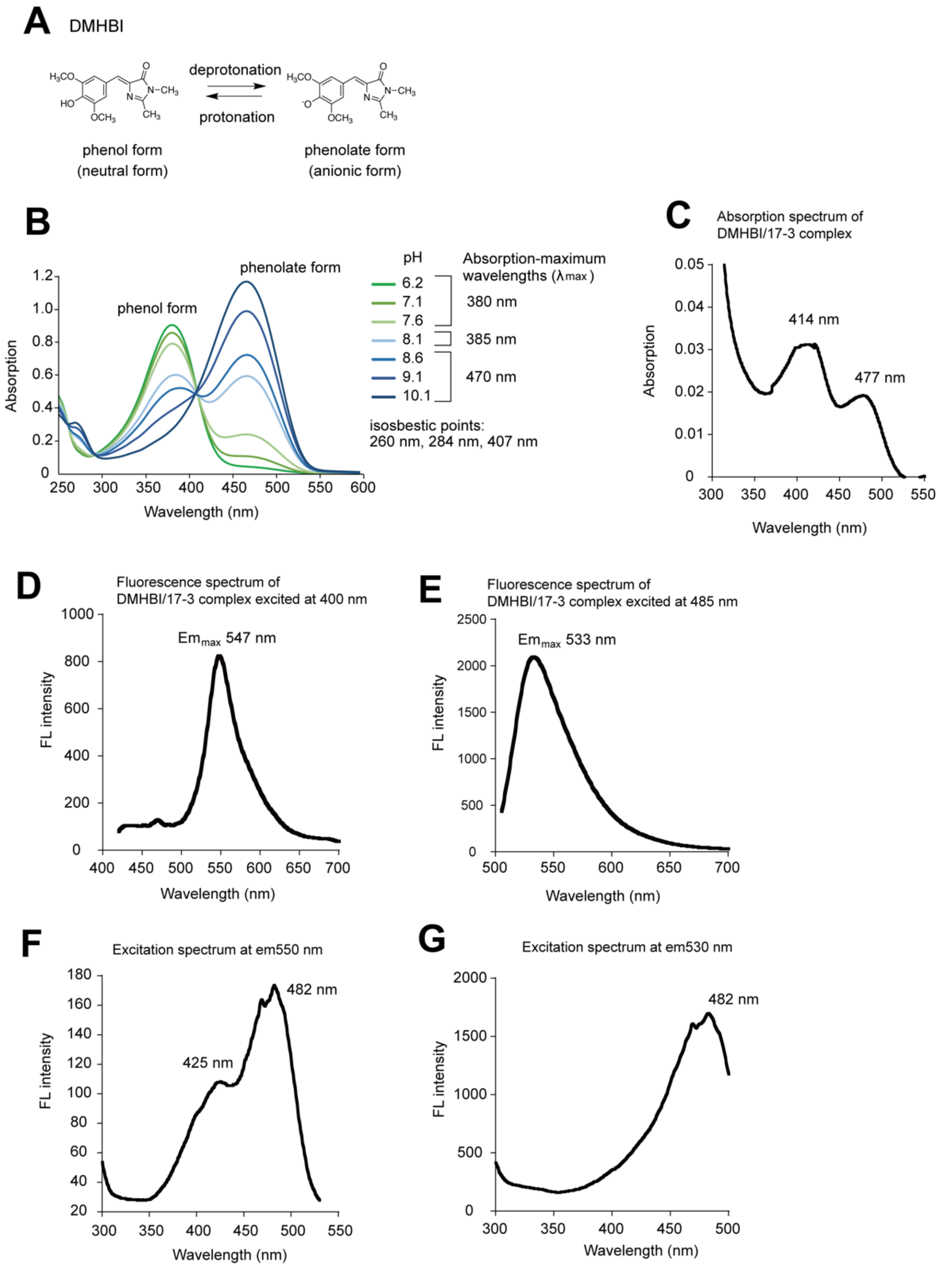

Figure S1. Photophysical properties of DMHBI and DMHBI/17-3 complex. (**A**) Neutral phenol form and anionic phenolate form of DMHBI. (**B**) pH-dependent changes in absorption spectrum of DMHBI. (**C**) Absorption spectrum of DMHBI complexed with 17-3 RNA. It is noted that both axes in panels B and C are different. (**D**, **E**) Fluorescence spectrum of DMHBI/17-3 RNA complex with excitation at 400 nm (D) and 485 nm (E). It is noted that both axes in panels D and E are different. (**F**, **G**) Excitation spectrum of DMHBI/17-3 RNA complex recorded with emission at 550 nm (F) and 530 nm (G). It is noted that both axes in panels F and G are different.

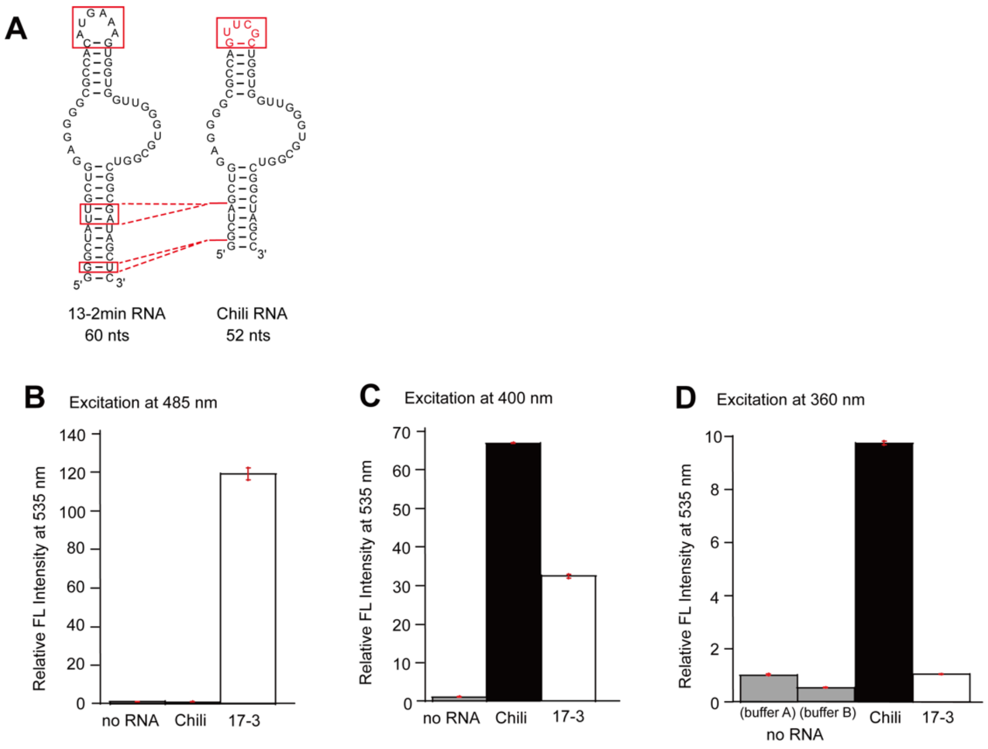

Figure S2. Comparison of fluorescent properties of DMHBI/17-3 RNA complex with DMHBI/Chili complex. (A) Sequences and secondary structures of 13-2min RNA and Chili (optimized 13-2min) RNA. (B, C, D) Relative fluorescence intensities of DMHBI/17-3 complex in buffer A (50 mM Tris-Cl pH 7.5 and 50 mM $MgCl_2$) and DMHBI/Chili complex in buffer B (40 mM Tris-Cl pH 7.5, 5 mM $MgCl_2$ and 100 mM KCl). It should be noted that the emission of DMHBI differed slightly in buffer A and buffer B with excitation at 360 nm (D). Emission of DMHBI was essentially the same in buffer A and buffer B with excitation at 485 nm (B) and 400 nm (C). Concentrations of RNA and DMHBI are 5 μM and 1 μM, respectively.

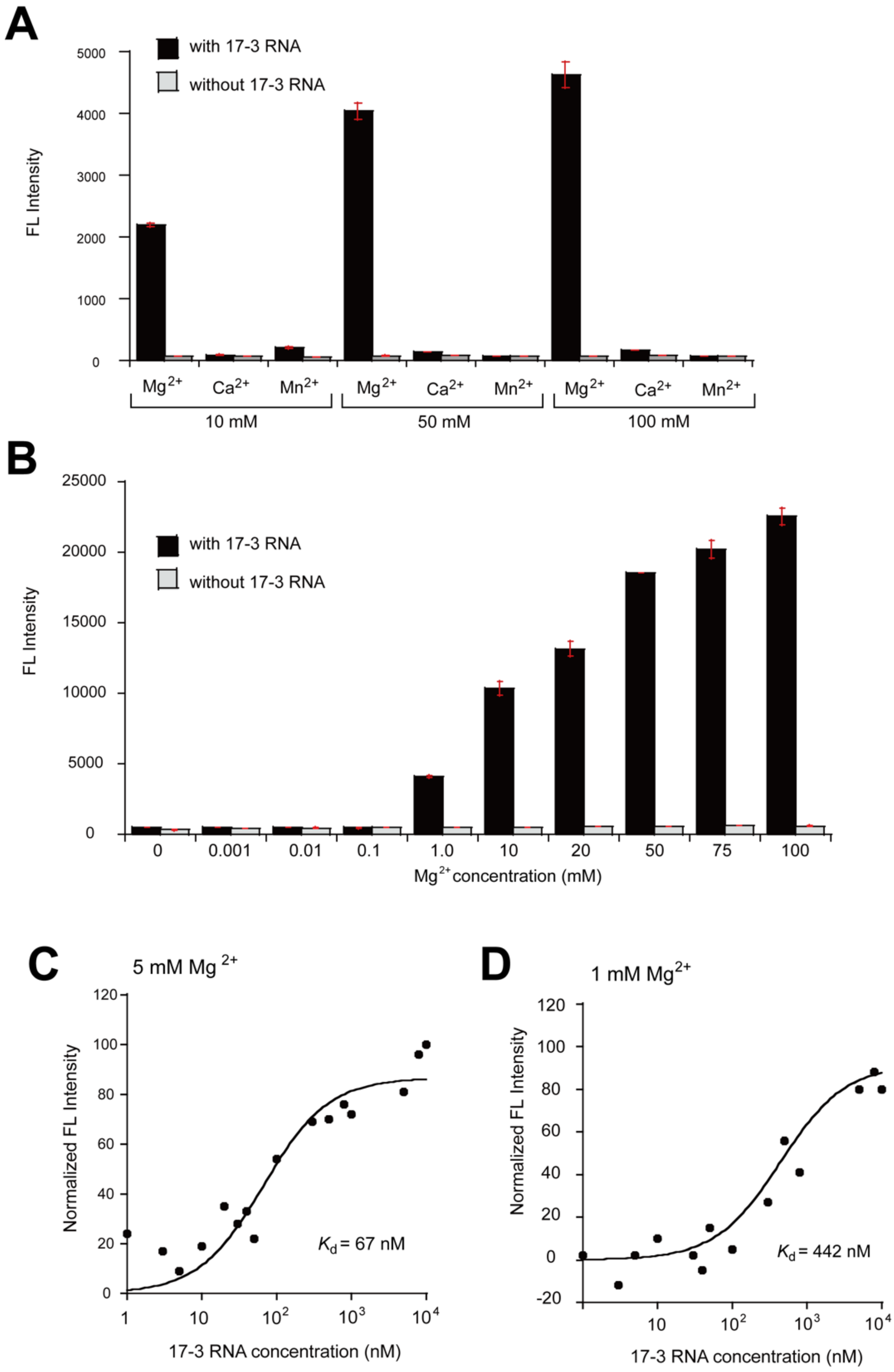

Figure S3. Fluorescent properties of the DMHBI/17-3 complex. (**A**) Fluorescent properties of DMHBI/17-3 complex with three different divalent ions. Concentrations of 17-3 RNA and DMHBI are both 0.1 µM. (**B**) Effects of Mg^{2+} concentration on the fluorescent properties of the DMHBI/17-3 complex under conditions of excess DMHBI. Concentrations of 17-3 RNA and DMHBI are 0.1 µM and 10 µM, respectively. (**C**, **D**) Estimation of the binding constant between 17-3 RNA and DMHBI in the presence of 5 mM Mg^{2+} (C) and 1 mM Mg^{2+} (D). The concentration of DMHBI is 5 nM.

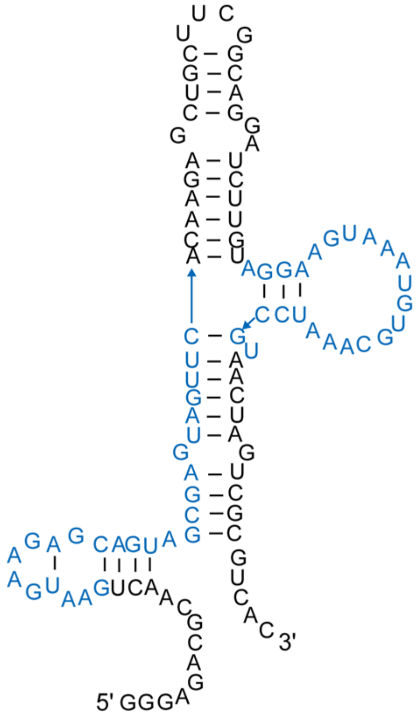

17-3 RNA structure
predicted by MXfold2

Figure S4. Secondary structure of 17-3 RNA predicted by MXFold2.

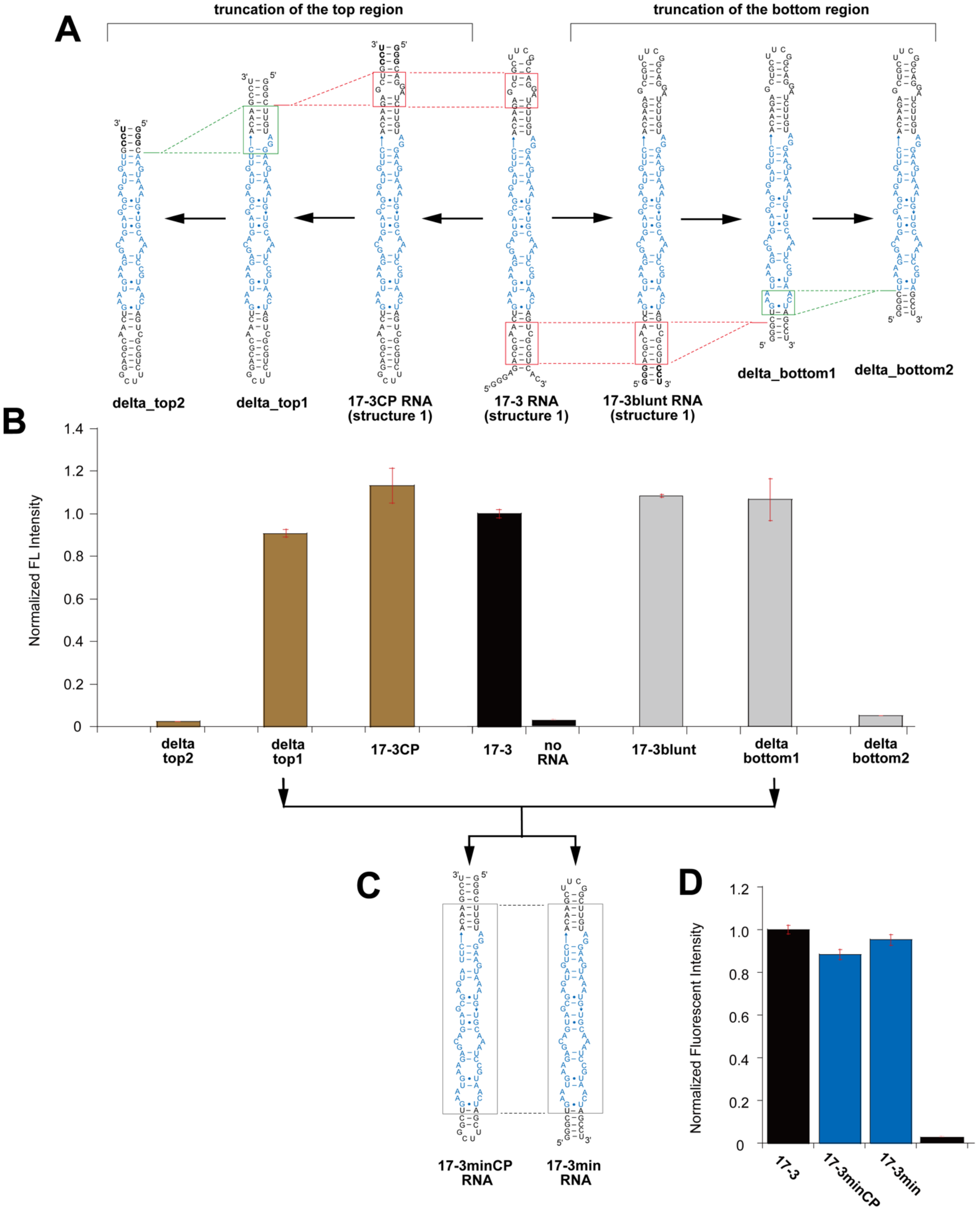

Figure S5. Truncation of 17-3 RNA. (**A**) Gradual truncation of the top and bottom regions of 17-3 RNA. (**B**) Fluorescent properties of truncated mutants of 17-3 RNA complexed with DMHBI. Concentrations of RNA, DMHBI, and Mg^{2+} are 0.1 μM, 0.1 μM, and 10 mM, respectively. (**C**) Secondary structures of minimized forms of 17-3 RNA retaining its fluorescent properties. (**D**) Relative fluorescent properties of 17-3min RNA and 17-3CPmin RNA.

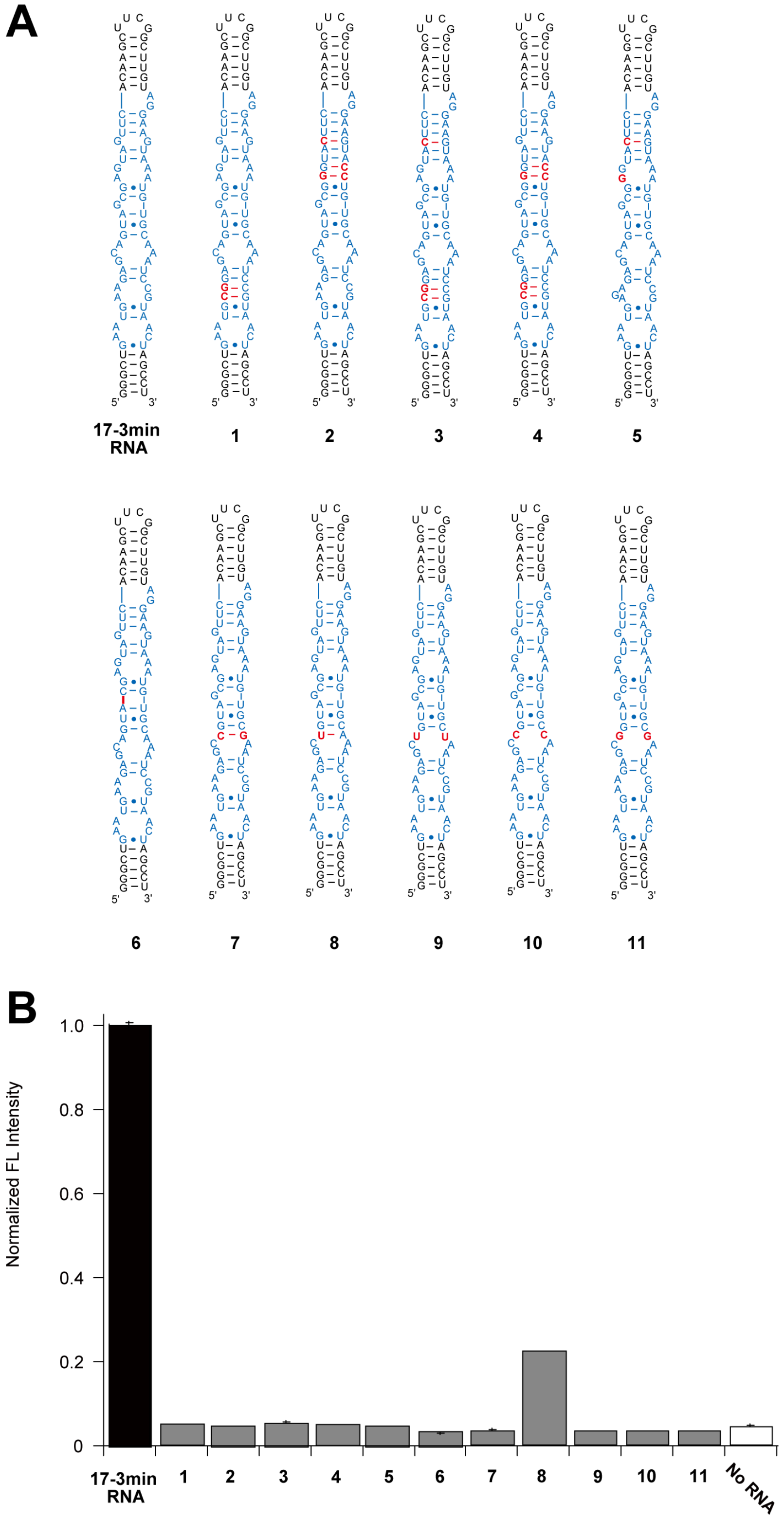

Figure S6. Evaluation of predicted structure 1 through base substitution mutations. (**A**) Sequences and secondary structures of a series of mutants of 17-3min RNA. (**B**) Relative fluorescent properties of a series of mutants of 17-3min RNA. Concentrations of RNA, DMHBI, and Mg^{2+} are 0.1 μM, 0.1 μM, and 10 mM, respectively.

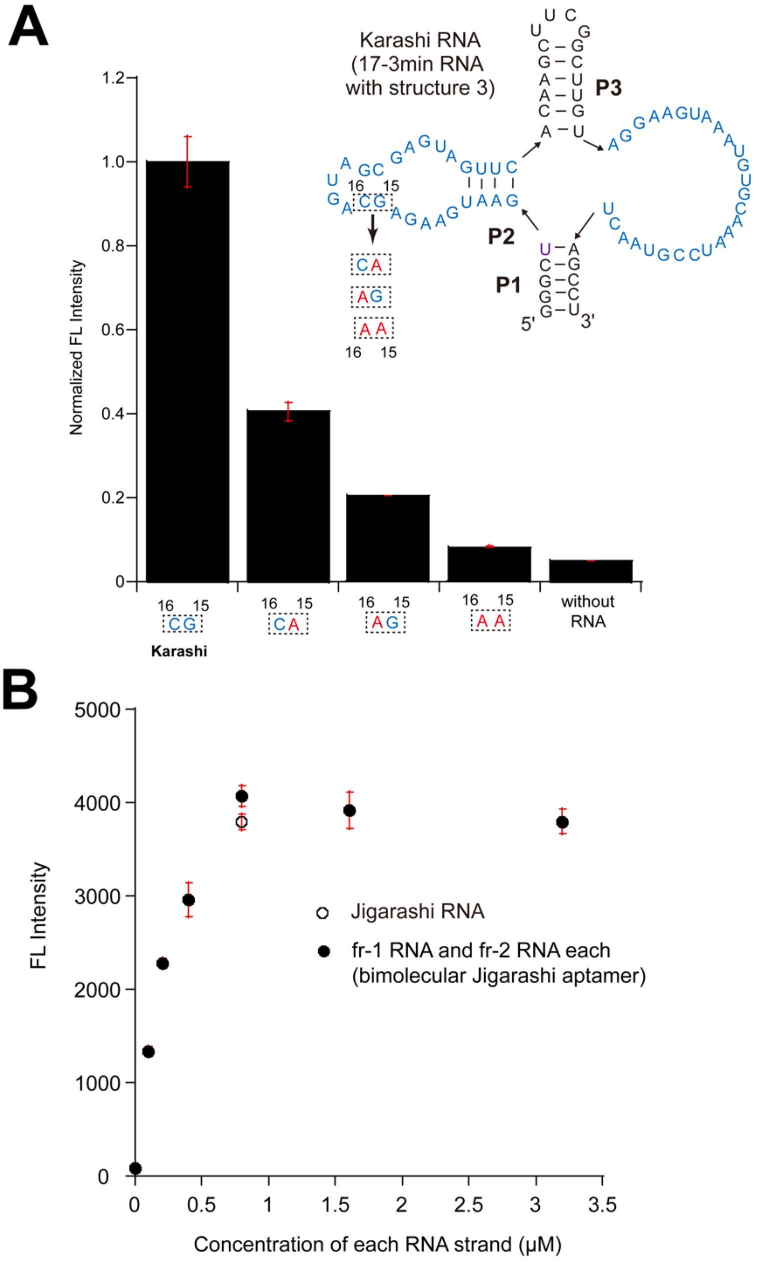

Figure S7. Evaluation of structure 3 of 17-3min RNA, named Karashi RNA. (**A**) Evaluation of the possible base pairs between positions 15, 16, and positions 21, 22. (**B**) Effects of RNA concentration on bimolecular version of Jigarashi RNA. Concentrations of DMHBI and Mg^{2+} are 0.1 μM and 50 mM, respectively.

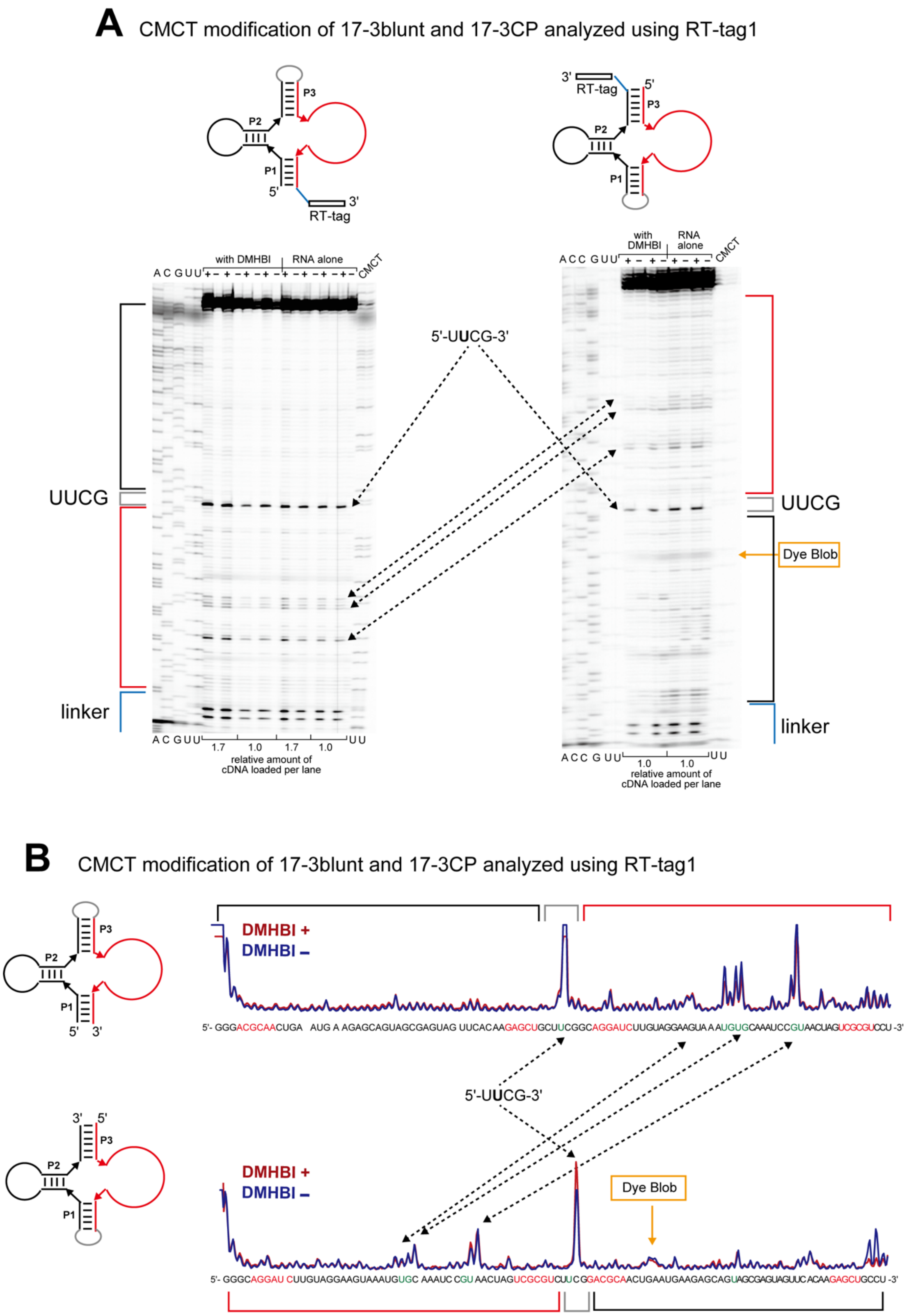

Figure S8. CMCT modification of 17-3blunt and 17-3CP. (**A**, **C**, **E**) Gel images of CMCT modification of 17-3blunt (left) and 17-3CP (right) detected by reverse transcription using RT-tag1 (A), RT-tag2 (C) or RT-tag3 (E). (**B**, **D**, **F**) CMCT modification patterns of 17-3blunt (top) and 17-3CP (bottom) detected by reverse transcription using RT-tag1 (A), RT-tag2 (C) or RT-tag3 (E).

C CMCT modification of 17-3blunt and 17-3CP analyzed using RT-tag2

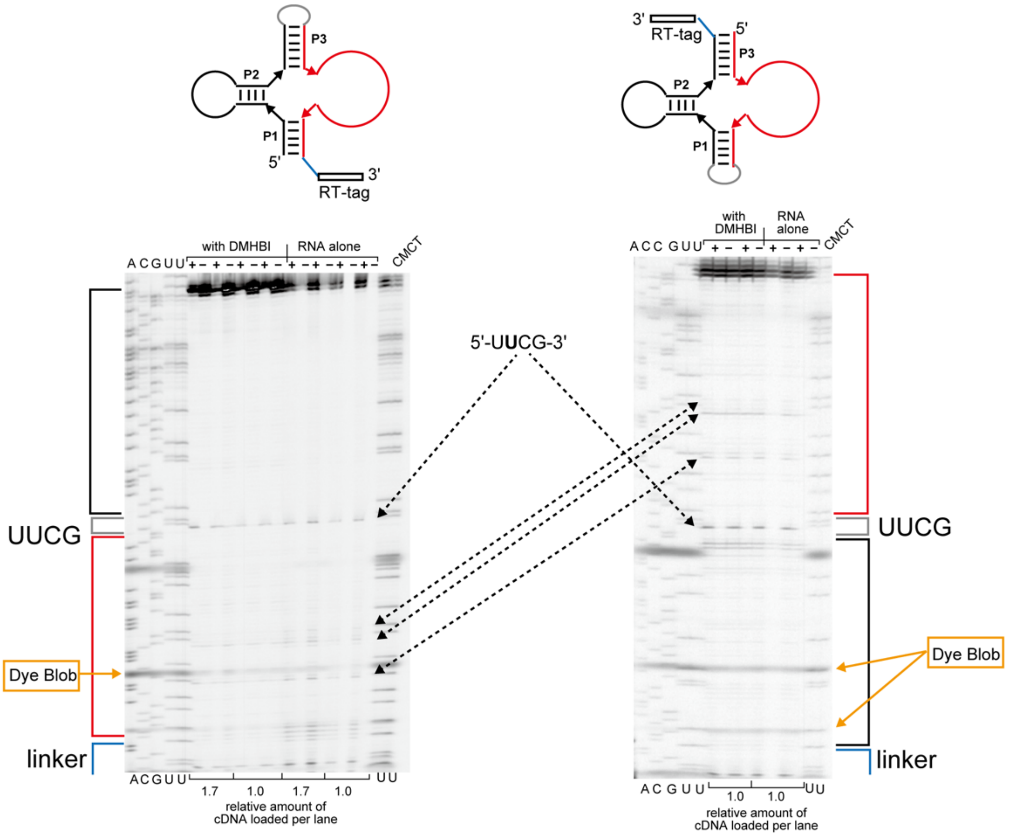

D CMCT modification of 17-3blunt and 17-3CP analyzed using RT-tag2

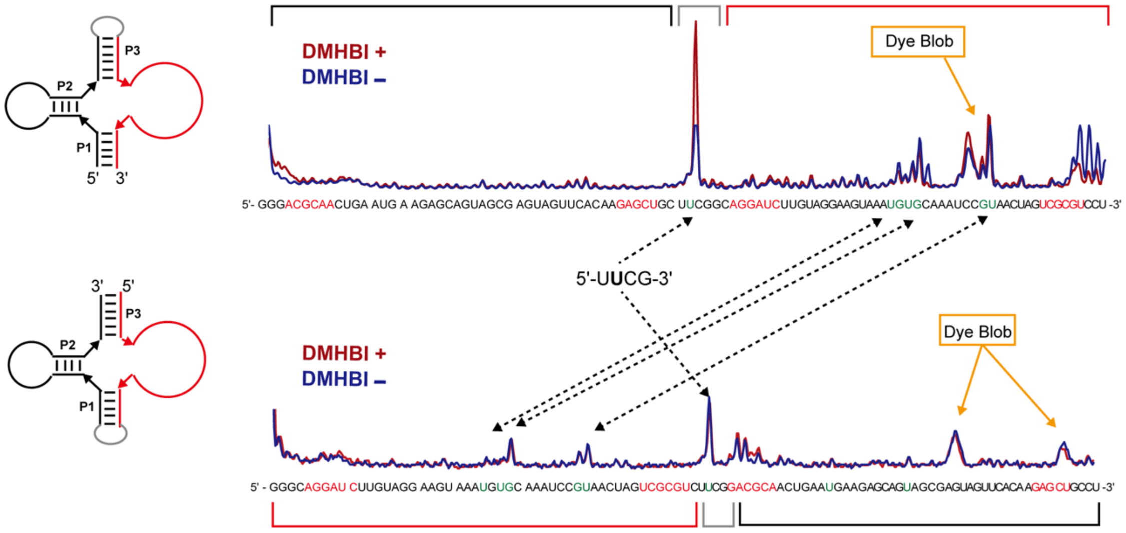

Figure S8. Continued.

E CMCT modification of 17-3blunt and 17-3CP analyzed using RT-tag3

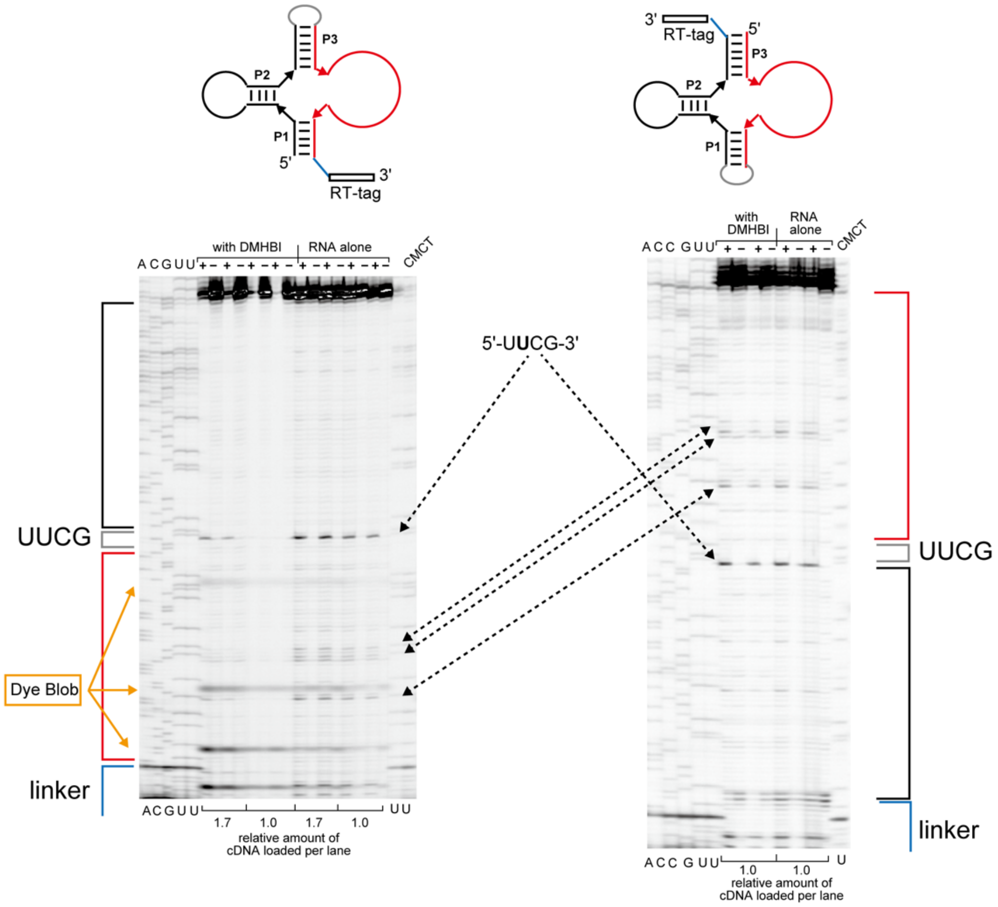

F CMCT modification of 17-3blunt and 17-3CP analyzed using RT-tag3

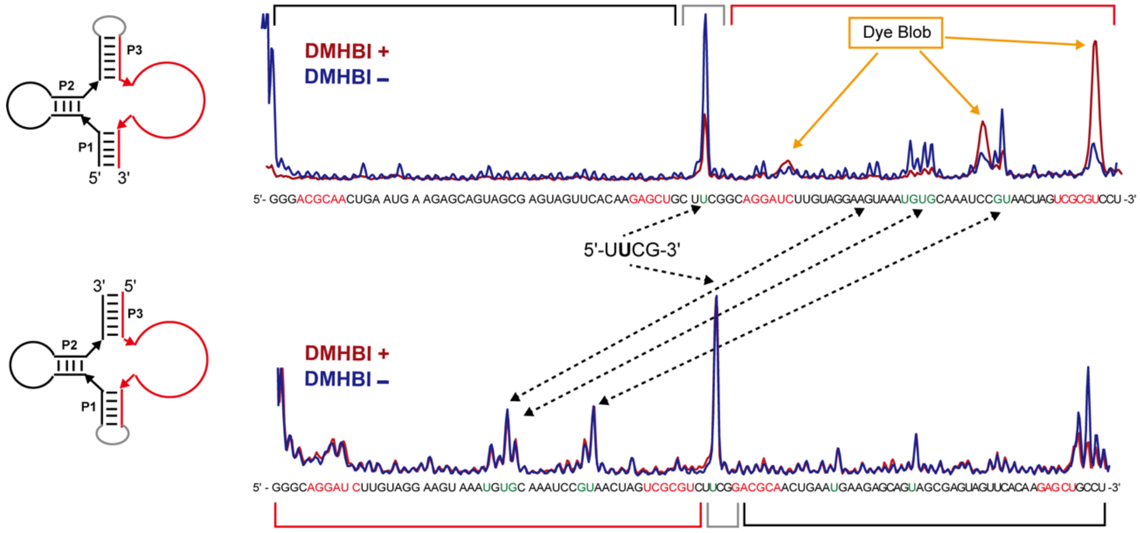

Figure S8. Continued.

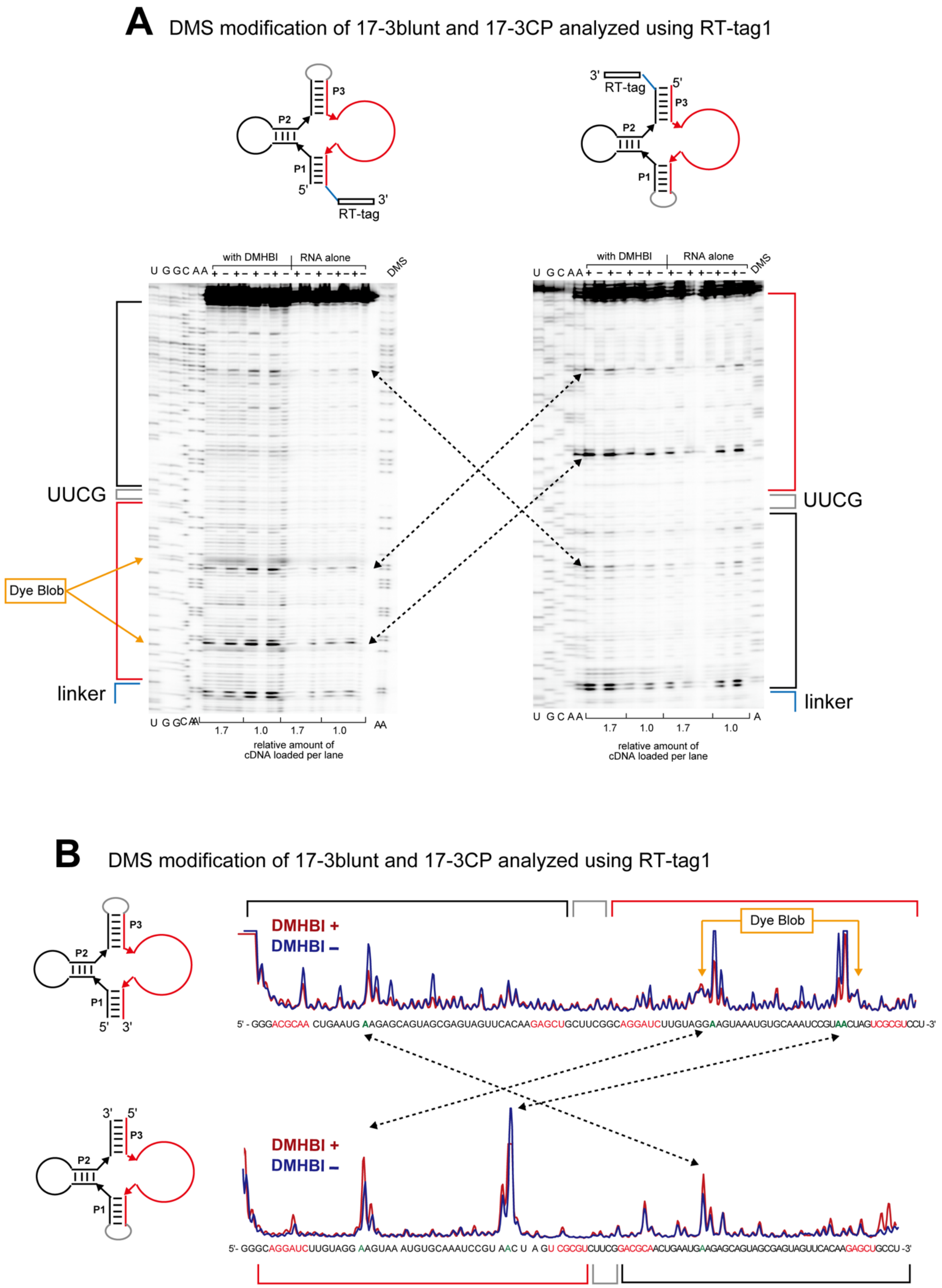

Figure S9. DMS modification of 17-3blunt and 17-3CP. (**A**, **C**, **E**) Gel images of DMS modification of 17-3blunt (left) and 17-3CP (right) detected by reverse transcription using RT-tag1 (A), RT-tag2 (C) or RT-tag3 (E). (B, **D**, **F**) DMS modification patterns of 17-3blunt (top) and 17-3CP (bottom) detected by reverse transcription using RT-tag1 (A), RT-tag2 (C) or RT-tag3 (E).

C DMS modification of 17-3blunt and 17-3CP analyzed using RT-tag2

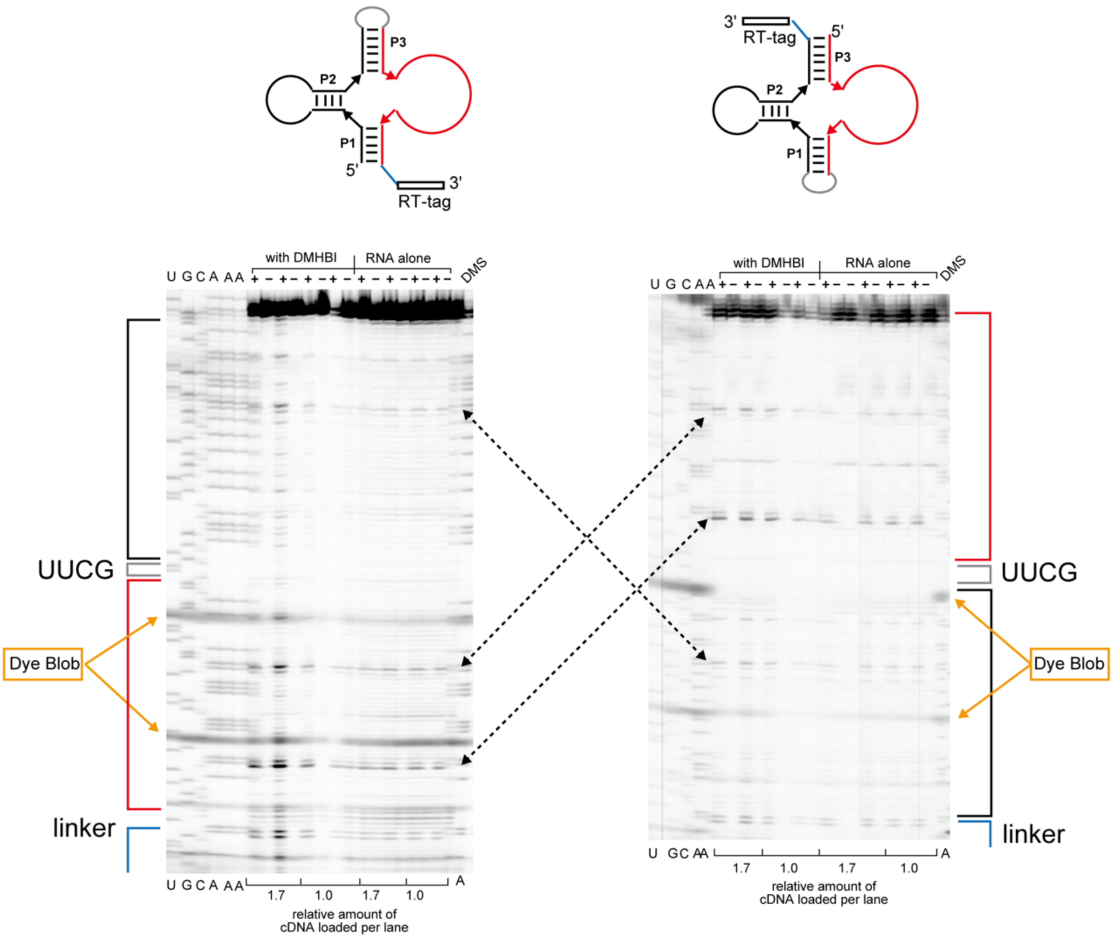

B DMS modification of 17-3blunt and 17-3CP analyzed using RT-tag2

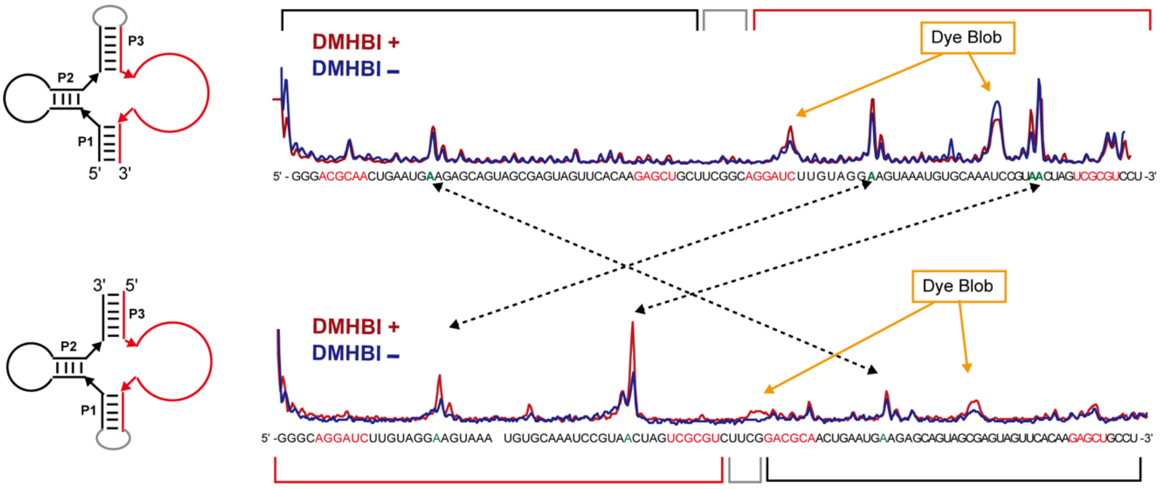

Figure S9. Continued.

E DMS modification of 17-3blunt and 17-3CP analyzed using RT-tag3

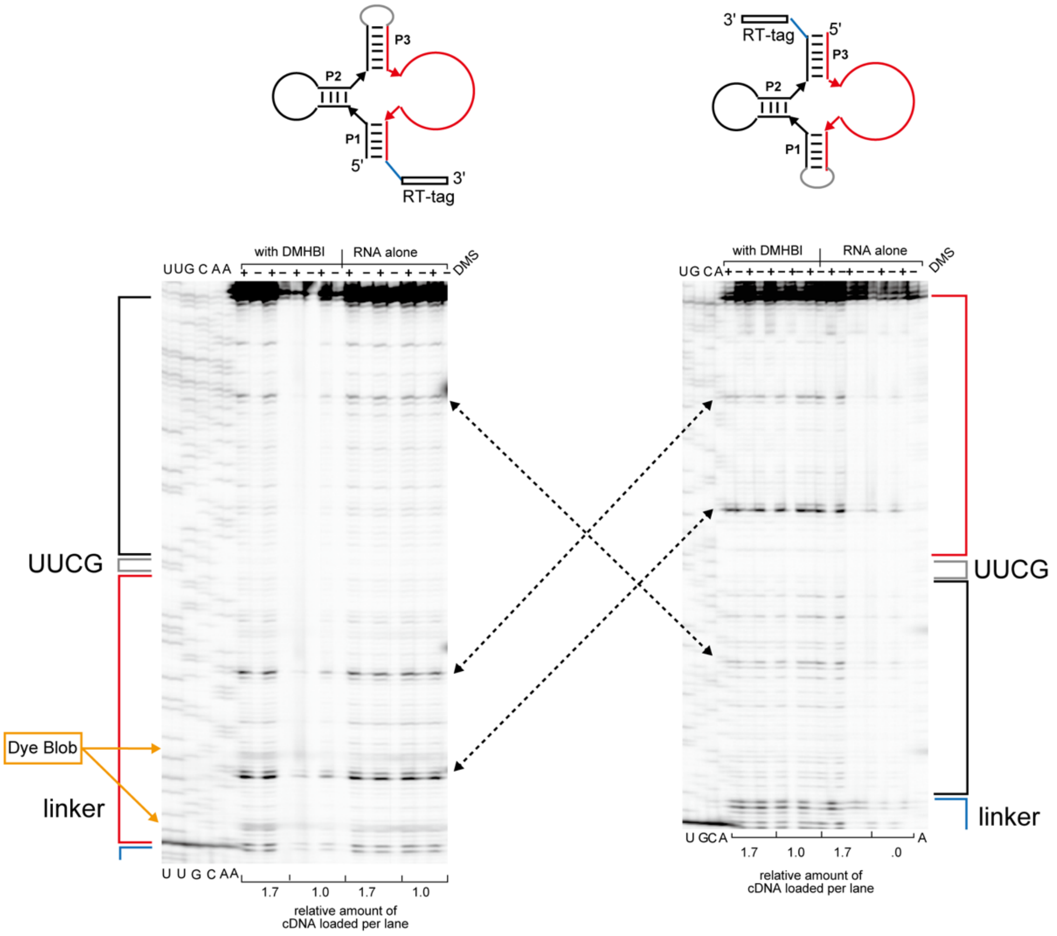

F DMS modification of 17-3blunt and 17-3CP analyzed using RT-tag3

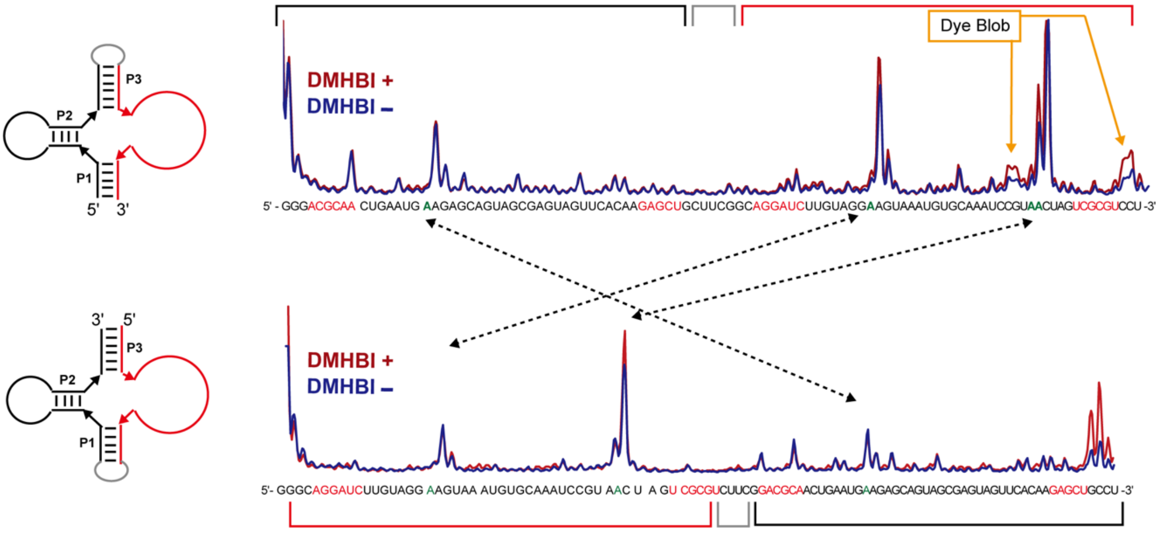

Figure S9. Continued.

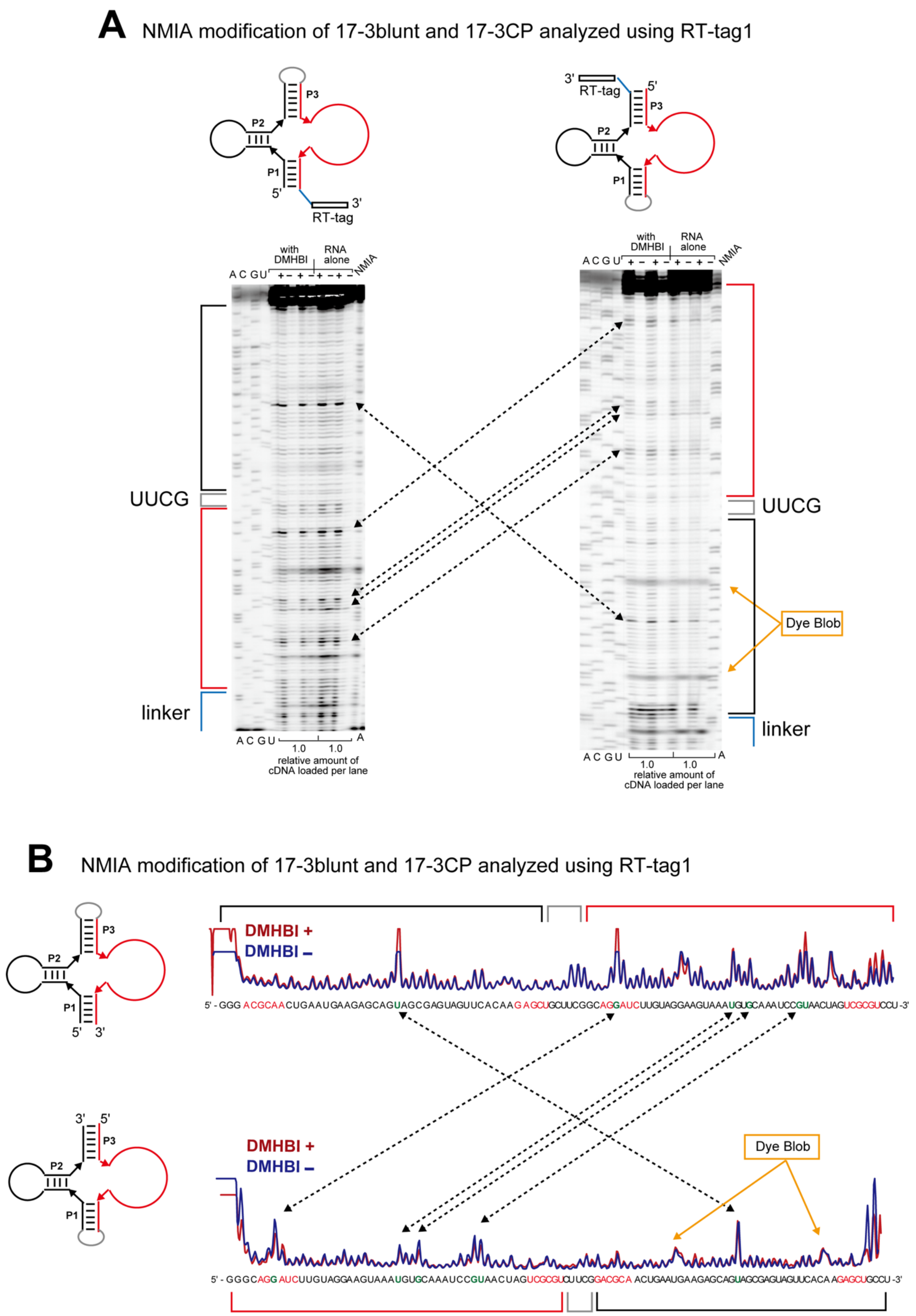

Figure S10. NMIA modification of 17-3blunt and 17-3CP. (**A**, **C**, **E**) Gel images of NMIA modification of 17-3blunt (left) and 17-3CP (right) detected by reverse transcription using RT-tag1 (A), RT-tag2 (C) or RT-tag3 (E). (**B**, **D**, **F**) NMIA modification patterns of 17-3blunt (top) and 17-3CP (bottom) detected by reverse transcription using RT-tag1 (A), RT-tag2 (C) or RT-tag3 (E).

C NMIA modification of 17-3blunt and 17-3CP analyzed using RT-tag2

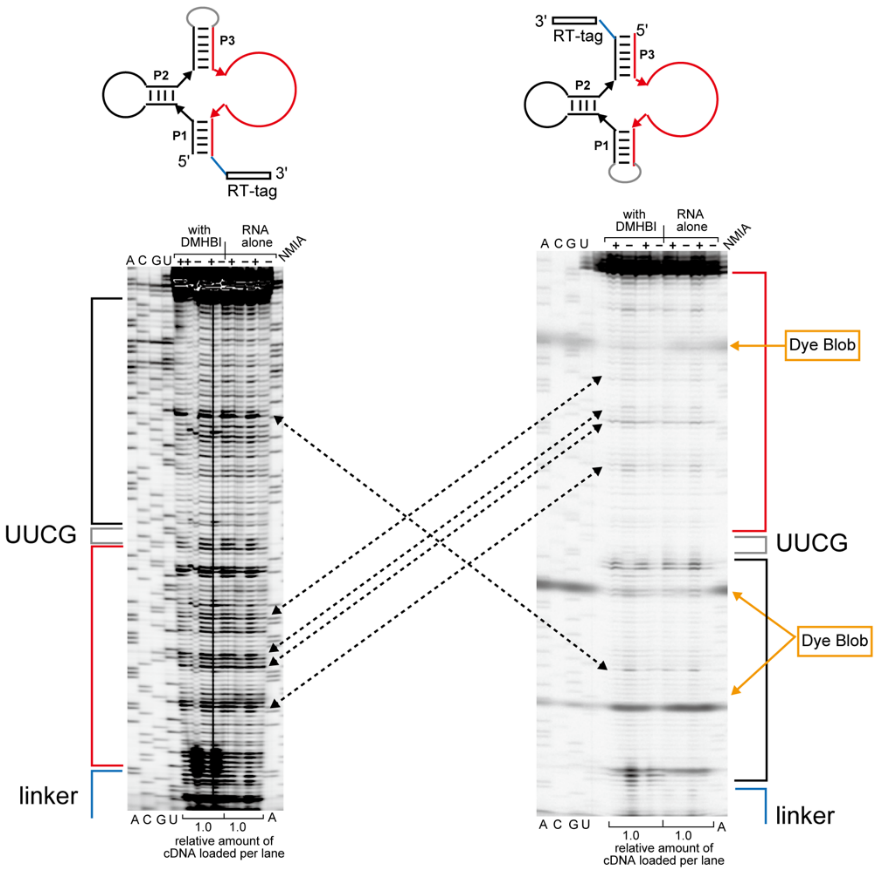

D NMIA modification of 17-3blunt and 17-3CP analyzed using RT-tag2

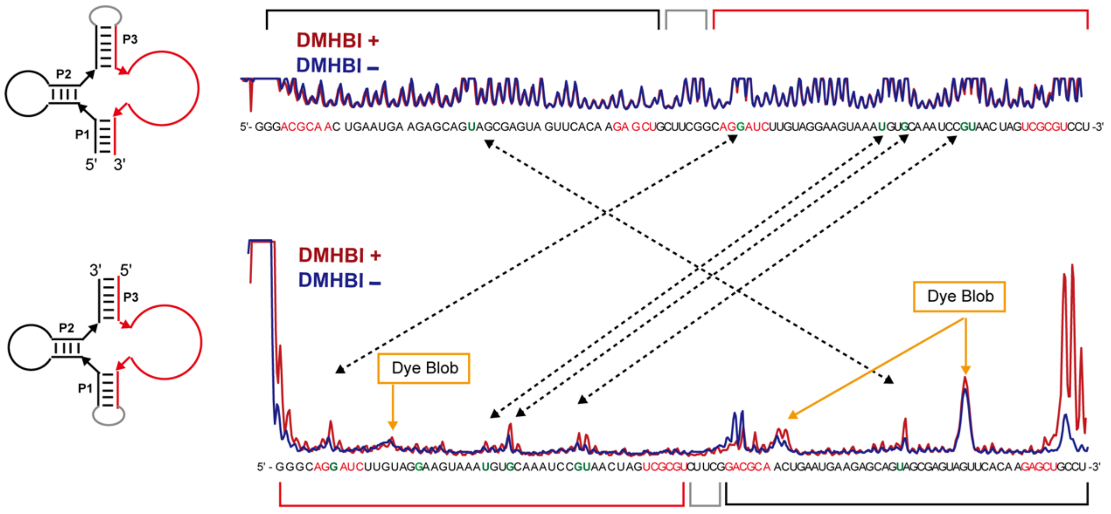

Figure S10. Continued.

E NMIA modification of 17-3blunt and 17-3CP analyzed using RT-tag3

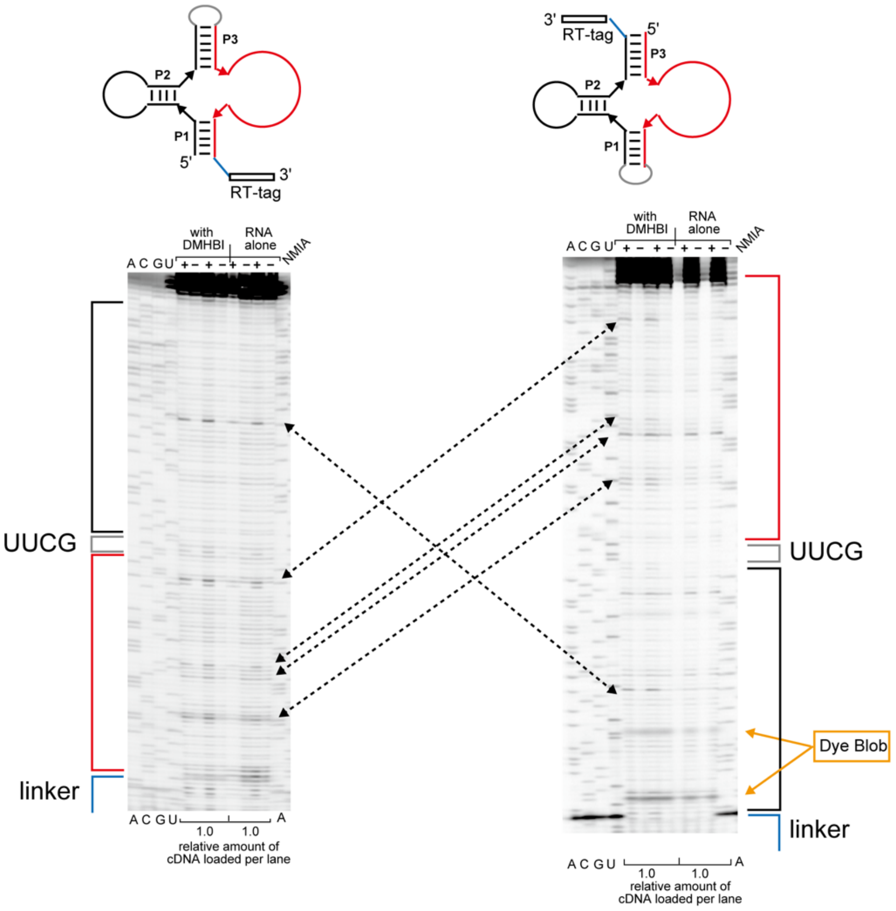

F NMIA modification of 17-3blunt and 17-3CP analyzed using RT-tag3

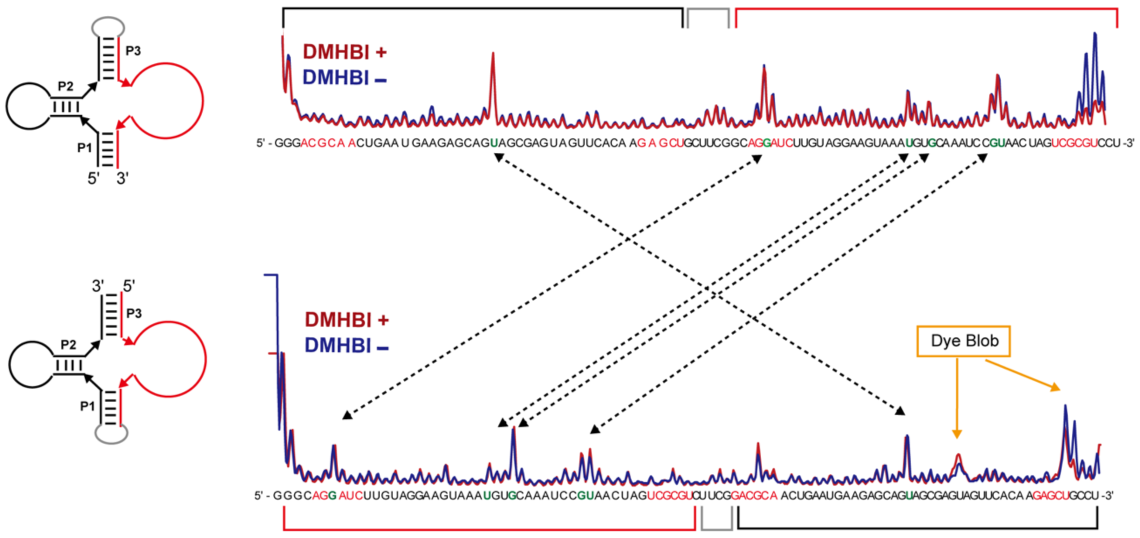

Figure S10. Continued.

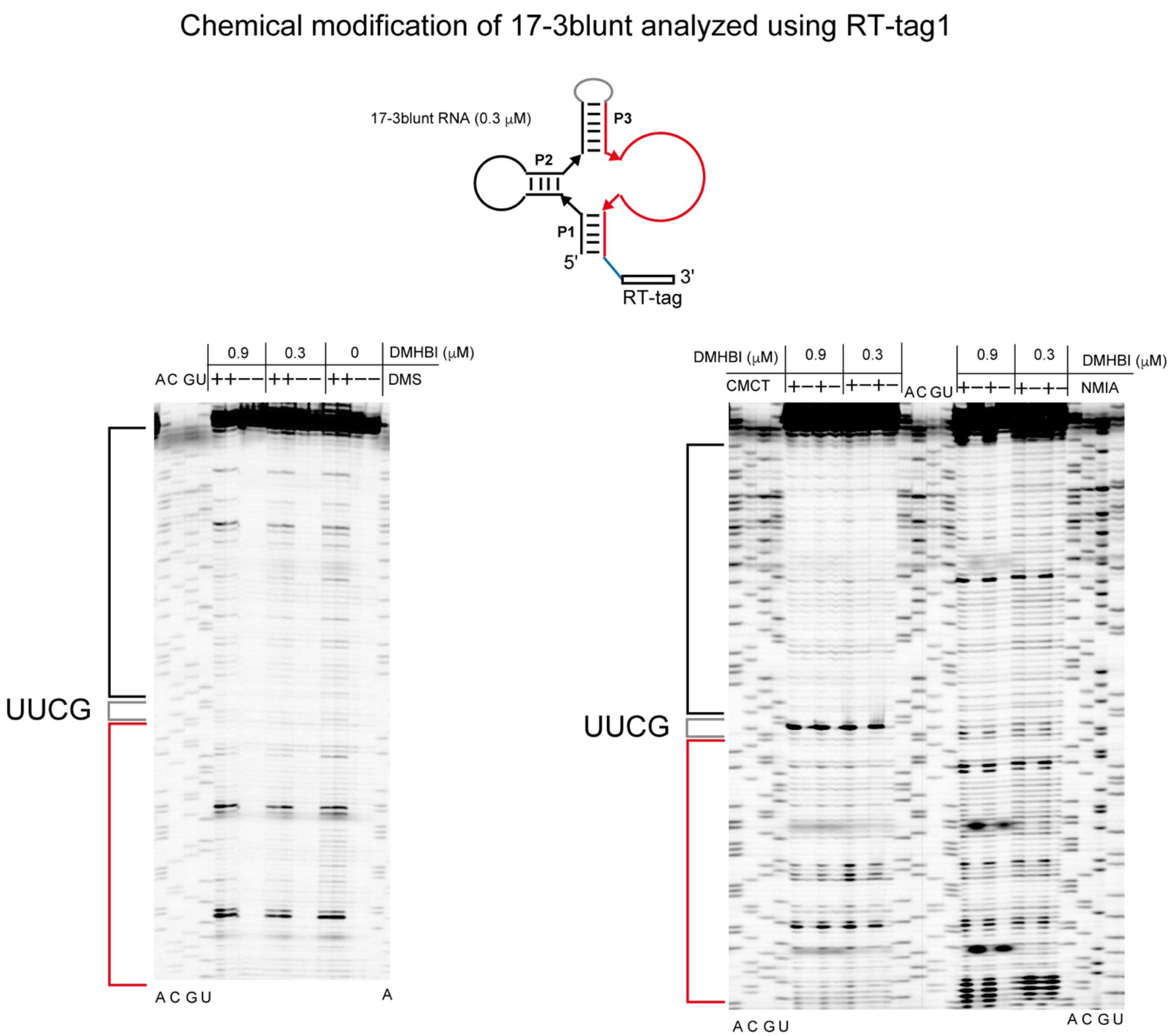

Figure S11. Chemical modification of 17-3blunt RNA (0.3 μM) in the presence of an equimolar amount (0.3 μM) or a three-fold excess (0.9 μM) of DMHBI. No significant changes in the modification patterns were observed between 0.3 μM and 0.9 μM DMHBI.

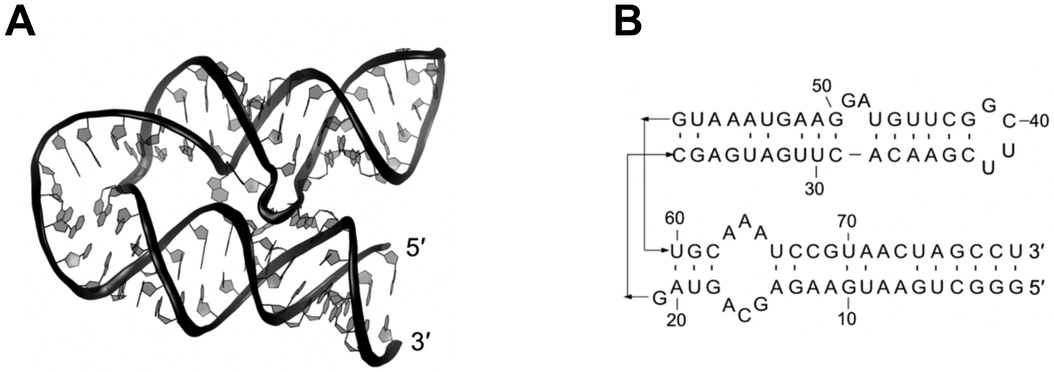

Figure S12. A 3D structure of 17-3min RNA predicted by AlphaFold3. (**A**) An apparently incorrect 3D structure of 17-3min RNA predicted by AlphaFold3. (**B**) The secondary structure of 17-3min RNA based on the apparently incorrect 3D structure predicted by AlphaFold3. This secondary structure is essentially the same as structure 1 of 17-3min RNA predicted by mfold.

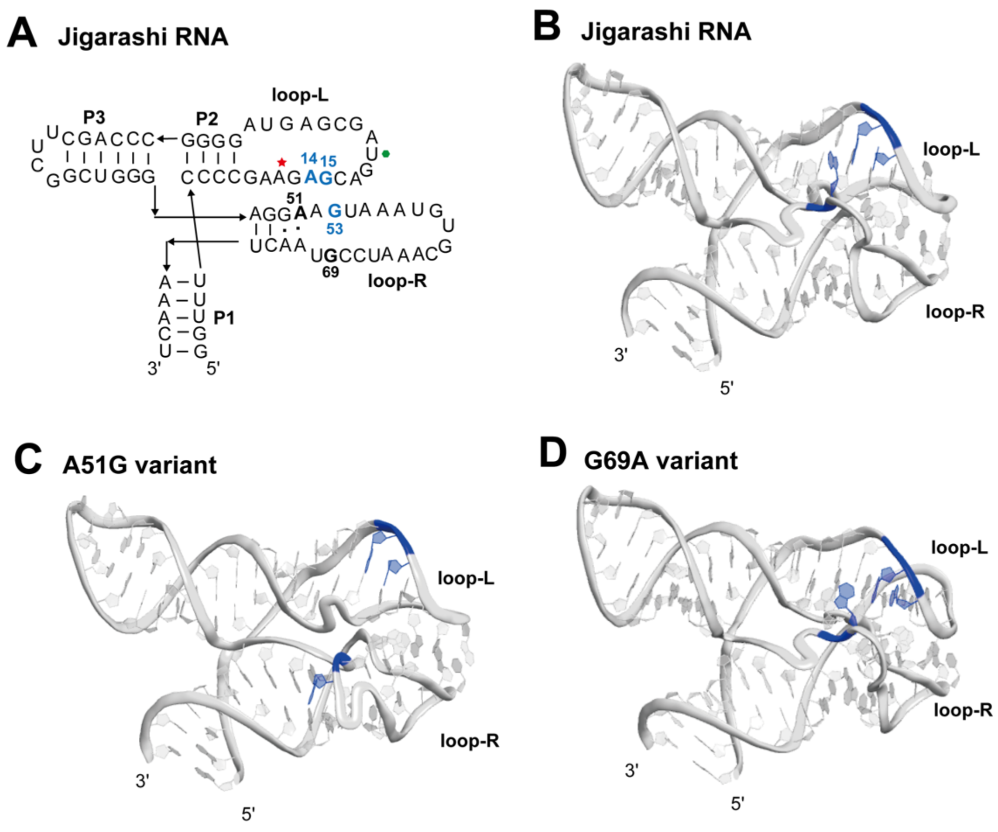

Figure S13. 3D structures of Jigarashi RNA and its variants predicted by AlphaFold3. (**A**) Secondary structures of Jigarashi RNA reflecting the rear side of its 3D structure predicted by AlphaFold3. (**B**, **C**, **D**) Rear of AlphaFold3 predicted 3D structures of Jigarashi RNA (B), its A51G variant (C), and its G69A variant (D). Positions A14, G15 and G53 are shown in blue.
